# Towards an asymmetric organocatalytic α-cyanation of β-ketoesters

**DOI:** 10.1016/j.tetlet.2015.02.116

**Published:** 2015-04-01

**Authors:** Raghunath Chowdhury, Johannes Schörgenhumer, Johanna Novacek, Mario Waser

**Affiliations:** Institute of Organic Chemistry, Johannes Kepler University Linz, Altenbergerstrasse 69, 4040 Linz, Austria

**Keywords:** Organocatalysis, Cinchona alkaloids, Hypervalent iodine, Phase-transfer catalysis

## Abstract

This communication describes the first proof of concept for an asymmetric α-cyanation of β-ketoesters using a hypervalent iodine-based electrophilic cyanide-transfer reagent. A series of different organocatalysts has been investigated and it was found that the use of naturally occurring Cinchona alkaloids allows obtaining the target products in good yields and with moderate enantioselectivities up to er = 76:24 under operationally simple conditions.

## Graphical abstract

**Figure:**
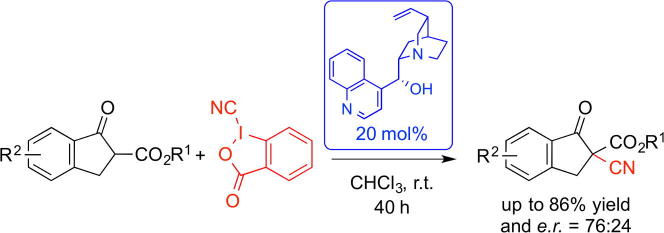

## Introduction

The construction of quaternary stereogenic centres is an important but challenging task.^1^ The use of hypervalent iodine compounds has emerged as a powerful method for the synthesis of highly functionalized target molecules over the last years.^2^ Some of the most important applications of hypervalent iodine reagents include the transfer of carbon electrophiles like aryl,^3^, ^4^ vinyl,^5^ alkynyl,^6^ or trifluoromethyl groups^7^ to prochiral nucleophiles. However, despite the spectacular progress made using these highly electrophilic reagents, their application in asymmetric reactions is still rather challenging, especially when it comes to the synthesis of quaternary stereogenic centres.^2^, ^3^, ^4^, ^5^, ^6^, ^7^ The main challenge that arises is to ensure stereocontrol in the C–C bond forming step with a suitable asymmetric catalyst. For example, it is nowadays well accepted that the α-arylation of β-ketoesters with diaryliodonium salts proceeds via enolate O-attack to the hypervalent iodine reagent first, followed by a [2,3]-rearrangement to create the α-stereogenic centre.3b However, the hereby formed primary addition product is a neutral species and therefore it is difficult to control the subsequent stereo-defining rearrangement with those asymmetric catalysts that are commonly used to control prochiral enolates (e.g., asymmetric ammonium salt catalysts^8^). The groups of MacMillan and Gaunt addressed this challenge by controlling the addition of silylenolates to diaryliodonium salts using chiral Cu(I)-based catalysts.(c), (d) One approach that allows for the successful combination of hypervalent iodine reagents and asymmetric organocatalysts^9^ is the use of benziodoxole derivatives as shown by the groups of Waser, Maruoka and Vesely for the asymmetric α-alkynylation of β-ketoesters^6a,b^, (b) or α-fluoro phenylsulfonyl nitromethane6c under phase-transfer catalysis. Hereby the primary addition product (O–I bond formation) is charged and therefore the chiral ammonium salt catalyst can control the subsequent rearrangement.^10^

**Scheme 1:**
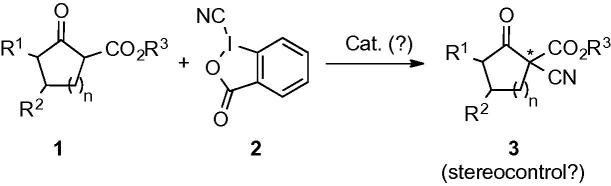
Targeted asymmetric α-cyanation of ketoesters **1**.

Inspired by these reports and based on our own interest in asymmetric phase-transfer catalysis^11^ we became interested in developing a protocol for the asymmetric α-cyanation of β-ketoesters **1** using the well-known cyano benziodoxole **2**^12^, ^13^, ^14^ as an electrophilic cyanide transfer reagent (Scheme 1).

## Results and discussion

Initial experiments were carried out using Cinchona alkaloid-based phase-transfer catalysts **A** for the reaction of *t*-butyl ester **1a** with cyanide **2**. During these initial investigations we obtained cyanation product **3a** accompanied by the formation of the α-brominated product **4** (see Table 1, entries 1–7 for representative results). The formation of α-halogenated products by reacting 1,3-dicarbonyl compounds and quaternary ammonium halides in the presence of hypervalent iodine reagents is a known transformation.^15^ While addressing this issue by screening different organocatalysts under different conditions we became aware of a very detailed and illustrative report by Chen et al. reporting the racemic cyanation of β-ketoesters **1** and the analogous amides using **2** as the cyanide transfer reagent under base-free conditions in different solvents.^13^ Interestingly, this group found that the α-hydroxy ketoester **5** is the only product under catalyst- and base-free conditions in solvents like toluene or CH_2_Cl_2_, whereas the use of DMF allowed them to totally suppress the formation of **5**, giving the α-cyano products **3** in excellent yields of around 90% within minutes at room temperature.^13^ In addition, they also observed that the presence of an achiral Cu(II) Lewis acid favours cyanation over hydroxylation to some extent when carrying out the reaction in CH_2_Cl_2_ (still <20% yield). Accordingly, the presence of a catalyst/additive significantly influences the product ratio in apolar solvents. This is also supported by the fact that **3a** was formed in every experiment when an organocatalyst was present but in neither case formation of **5** was observed (by ^1^H NMR of the crude mixture).

**Table 1:** Identification of the most active organocatalyst and best-suited reaction conditions for the asymmetric synthesis of **3a**

**Figure:**
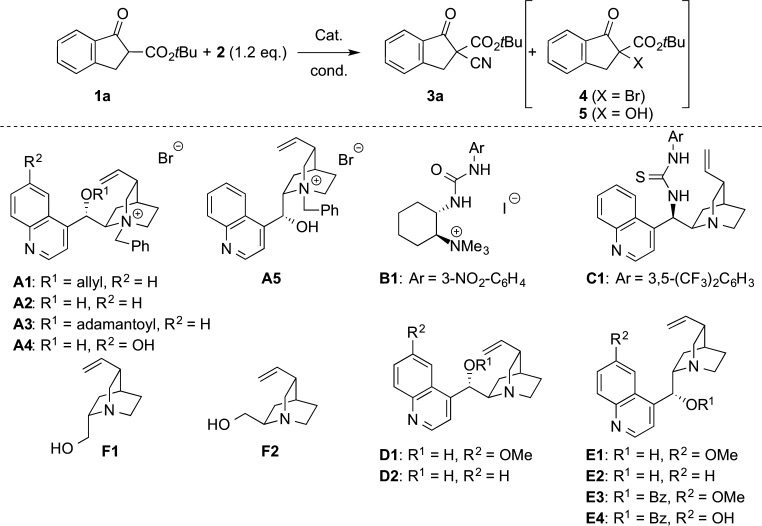

Entry	Cat. (mol %)	Solvent	Base	*T* (°C)	*t* (h)	**3a**^a^ (%)	**4**^b^ (%)	er^c^ (±)
1	**A1** (20%)	CH_2_Cl_2_	K_2_CO_3_ (5 equiv)	25	24^d^	58	10–15	48:52
2	**A1** (20%)	CH_2_Cl_2_	—	25	24	60	15–20	48:52
3	**A2** (20%)	CH_2_Cl_2_	—	25	24^d^	30	15–20	55:45
4	**A3** (20%)	CH_2_Cl_2_	—	25	24	58	15–20	50:50
5	**A4** (20%)	CH_2_Cl_2_	—	25	24^d^	20^b^	15–20	50:50
6	**A5** (20%)	CH_2_Cl_2_	—	25	24^d^	39	20 (17)^a^	42:58
7	**A5** (20%)	CH_2_Cl_2_	K_2_CO_3_ (5 equiv)	25	24^d^	47	15–20 (15)^a^	44:56
8	**B1** (10%)	CH_2_Cl_2_	—	25	40^d^	39	n.d.	37:63
9	**C1** (20%)	Toluene	—	25	40^d^	25	n.d.	34:66
10	**D1** (20%)	CH_2_Cl_2_	—	25	40	80	n.d.	64:36
11	**D2** (20%)	CH_2_Cl_2_	—	25	40	78	n.d.	66:34
12	**E1** (20%)	CH_2_Cl_2_	—	25	40	80	n.d.	38:62
13	**E2** (20%)	CH_2_Cl_2_	—	25	40	83	n.d.	30:70
14	**E3** (20%)	CH_2_Cl_2_	—	25	40	79	n.d.	50:50
15	**E4** (20%)	CH_2_Cl_2_	—	25	40	70	n.d.	42:58
16	**F1** (20%)	CH_2_Cl_2_	—	25	40^d^	66	n.d.	50:50
17	**F2** (20%)	CH_2_Cl_2_	—	25	40^d^	67	n.d.	47:53
18	**E2** (20%)	Toluene	—	25	40^d^	62	n.d.	33:67
19	**E2** (20%)	MTBE	—	25	40	78	n.d.	41:59
20	**E2** (20%)	CHCl_3_	—	25	40	70^e^	n.d.	26:74
21	**E2** (10%)	CHCl_3_	—	25	40^d^	34	n.d.	26:74
22	**E2** (5%)	CHCl_3_	—	25	40^d^	32	n.d.	34:66
23	**E2** (40%)	CHCl_3_	—	25	40	85	n.d.	30:70
24	**E2** (20%)	CHCl_3_	—	0	72^d^	52	n.d.	25:75
25	**E2** (20%)	CHCl_3_	—	40	40	57	n.d.	27:73

a Isolated yield.

b Determined by ^1^H NMR of the crude reaction product.

c Determined by HPLC using a chiral stationary phase.

d Less than 90% conversion of **1a**.

e Using 2 equiv of **2** gave **3a** in 75% yield and the same enantioselectivity.

Table 1 gives an overview of the most significant results obtained in a detailed screening of catalysts and reaction conditions. Initial experiments with phase-transfer catalysts **A** revealed that **3a** can be obtained with low enantioselectivities when using free-OH containing catalysts **A2** or **A5** and it also turned out that base-free and basic conditions give almost the same selectivity (see entries 6 and 7). Cyanation was always the dominant reaction but it should be pointed out that conversion of starting material was usually not complete within 24 h and formation of racemic bromide side-product **4** was always observed (more or less quantitatively with respect to the amount of ammonium bromide catalyst used). As the presence of an H-bonding donor^16^ favoured selectivity, we next tested urea-containing ammonium salt **B** that was recently introduced by our group.^17^ The enantioselectivity could be slightly enhanced but the yield was not very high (entry 8). Thus, no further screening of quaternary ammonium salts was carried out but we tested different *tert*-amine containing bifunctional H-bonding donors next. Use of the thiourea-containing **C1** resulted in a slightly higher selectivity of 66:34 (entry 9) but the yield was relatively low even after 40 h. In contrast, the use of simple Cinchona alkaloids **D** and **E** resulted in high yields and moderate enantioselectivities when using 20 mol% of catalyst in CH_2_Cl_2_ at room temperature (40 h reaction time ensured more than 90% conversion) (entries 10–13). Interestingly, cinchonidine (**E2**) gave **3a** with the highest selectivity among the four tested naturally occurring Cinchona alkaloids (entry 13). Blocking of the 9-OH group totally suppressed chiral induction (**E3**, entry 14), whereas use of the 6′-OH containing catalyst **E4** gave **3a** with reduced selectivity compared to the use of **E2**. Testing the truncated aminoalcohols **F** resulted in racemic **3a** only, indicating that the quinoline moiety plays an important role. Again, in neither case the formation of **5** was observed when using such different aminoalcohols as catalysts. Screening of different solvents showed that CHCl_3_ is best-suited to obtain enantioenriched **3a** in reasonable yield and with moderate selectivity (er = 74:26; entry 20). Unfortunately neither a change in temperature, nor dilution of the reaction mixture (not given in the table) or a change in the stoichiometric ratio of **1** and **2** had a beneficial effect. Lowering the amount of catalyst resulted in a lower conversion rate and a lower selectivity when using 5 mol % catalyst (entries 21 and 22). On the other hand increasing the catalyst loading also caused a slight decrease of enantioselectivity (entry 23). By following the enantioselectivity in dependence of the reaction progress we found no noteworthy trend, thus degradation of the catalyst as well as formation of a more active catalyst species during the reaction can be excluded at the present stage of knowledge about this transformation.

**Scheme 2:**
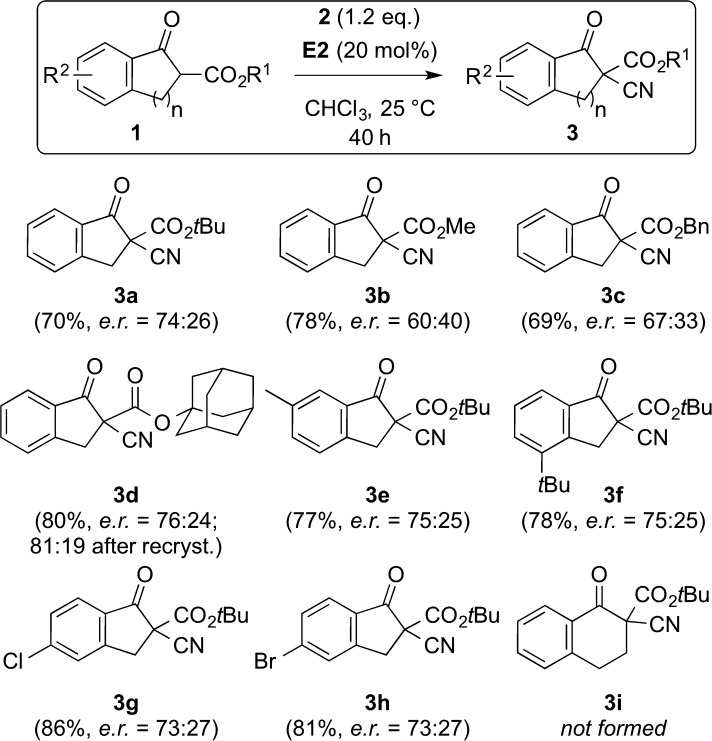
Application scope of the asymmetric α-cyanation of **1** (in each case the (−)-enantiomer was the major stereoisomer).

Based on this detailed catalyst screening, which revealed cinchonidine (E2) to be the best-suited chiral aminoalcohol catalyst to obtain α-cyano ketoester **3a** with a modest enantioselectivity of 74:26 in good yield, we next tested the scope of this protocol for some differently substituted β-ketoesters **1** (Scheme 2). The nature of the ester group had a strong influence on the observed enantioselectivity. While the methyl and benzylesters **3b** and **3c** could only be obtained with rather low selectivity, the adamantyl ester **3d** could be accessed with an er of 76:24 under the standard conditions. Unfortunately, attempts to increase the enantiopurity by recrystallization were not very successful (best er for **3d** was 81:19 but in low yield only). In contrast to the strong influence of the ester group, different aryl substituents did not significantly influence the outcome. On the other hand, tetralone derivative **3i** could not be accessed under these reaction conditions (this compound is however accessible when carrying out the reaction under the racemic conditions reported previously by Chen et al.^13^).

## Conclusion

A first proof of concept towards an asymmetric α-cyanation of β-ketoesters using a hypervalent iodine-based cyanide-transfer reagent has been made. The use of naturally occurring Cinchona alkaloids as chiral organocatalysts allowed us to obtain the target products in good yields and with moderate enantioselectivities (up to er = 76:24) under operationally simple conditions. Noteworthy, the presence of catalysts/additives significantly influences the product ratio (cyano vs hydroxyl transfer) in this approach (see also Ref. 13). Despite the obtained enantioselectivity is not very high yet, the herein reported protocol should be promising with respect to further development and future studies will aim on the identification of more selective catalysts (e.g., designed chiral aminoalcohols or asymmetric transition metal catalysts) and on the expansion of this concept towards other (prochiral) nucleophiles.

## Experimental section

### General procedure for the α-cyanation of β-ketoesters **1**

Cyano benziodoxole **2** (1.2 equiv) was added to a stirred solution of the corresponding β-ketoester **1** and cinchonidine (**E2**, 20 mol %) in chloroform (5 mL per mmol **1**) at room temperature. The reaction mixture was stirred at this temperature for 40 h. The crude product was directly transferred to a silica-gel column and eluted with a gradient of heptane and EtOAc to give the products **3** in the reported yields and enantiopurities.

*Cyanide 3a*: Obtained in 70% (0.7 mmol scale) as an oil that crystallizes upon prolonged storage in the refrigerator. Mp: 31–32 °C; [*α*]_D_^20^ (*c* 1.2, DCM, er = 74:26) = −15; ^1^H NMR (300 MHz, *δ*, CDCl_3_, 298 K): 7.83 (d, *J* = 7.8 Hz, 1H), 7.68–7.73 (m, 1H), 7.44–7.53 (m, 2H), 3.87 (d, *J* = 17.4 Hz, 1H), 3.63 (d, *J* = 17.4 Hz, 1H), 1.48 (s, 9H) ppm; ^13^C NMR (75 MHz, *δ*, CDCl_3_, 298 K): 191.3, 162.8, 151.7, 136.8, 132.3, 128.8, 126.4, 126.2, 116.1, 85.9, 55.2, 37.5, 27.6 (3C) ppm; IR (film): $\overset{¯}{\nu }$ = 2977, 2935, 2245, 1723, 1148, 835, 735 cm^−1^; HRMS (ESI): *m*/*z* calcd for C_15_H_15_NO_3_: 280.0960 [M+Na]^+^; found: 280.0946. The enantioselectivity was determined by HPLC (Chiralcel OD-H, eluent: hexane:*i*-PrOH = 90:10, 0.5 mL/min, 10 °C, retention times: *t_minor_* = 15.7 min, *t_major_* = 16.7 min).

## Supplementary data

Supplementary data General procedure and spectral data.
