# Supplementary material for: Towards an asymmetric organocatalytic α-cyanation of β-ketoesters
Source: Tetrahedron Lett. 2015 Apr 1;56(14):1911–4. doi: 10.1016/j.tetlet.2015.02.116 (PMC4366011; doi:10.1016/j.tetlet.2015.02.116)
Supplement: Supplementary data — General procedure and spectral data. [file mmc1.pdf]

SUPPORTING INFORMATION

Towards an asymmetric organocatalytic α-cyanation of β-ketoesters

Raghunath Chowdhury, Johannes Schörgenhumer, Johanna Novacek, and Mario Waser*

Institute of Organic Chemistry, Johannes Kepler University Linz, Altenbergerstraße 69, 4040 Linz, Austria. Fax: +43 732 2468 8747; Tel: +43 732 2468 8748;

E-mail: Mario.waser@jku.at; raghuch14@gmail.com

1. General Information: 2
2. Asymmetric α-Cyanation of β-Ketoesters: 3
3. Copies of NMR spectra: 7
4. HPLC Chromatograms: 16

1. General Information:

^{1}H- and ^{13}C-NMR spectra were recorded on a Bruker Avance III 300 MHz spectrometer and on a Bruker Avance III 700 MHz spectrometer with TCI cryoprobe. All NMR spectra were referenced on the solvent peak. High resolution mass spectra were obtained using a Thermo Fisher Scientific LTQ Orbitrap XL with an Ion Max API Source. IR spectra were recorded on a Bruker Tensor 27 FT-IR spectrometer with ATR unit. Optical rotations were recorded on a Schmidt + Haensch Polarimeter Model UniPol L 1000. HPLC was performed using a Dionex Summit HPLC system with a Chiralcel OD-H (250 x 4.6 mm) or a Chiralpak AD-H (250 x 4.6 mm, 5 μm) chiral stationary phase. All chemicals were purchased from commercial suppliers and used without further purification unless otherwise stated. All reactions were performed under an Ar-atmosphere. Starting β-ketoesters **1** were either purchased from commercial suppliers or prepared according to literature-known methods.[1] 1-Cyano-1,2-benziodoxol-3(1*H*)-one (**2**) was prepared according to known literature procedure.[2,3] Cinchona alkaloids were obtained from commercial suppliers. Toluene and MTBE were distilled from Na under Ar atmosphere. Dichloromethane and Chloroform were distilled from CaH_2 under Ar atmosphere. Racemic products were synthesized according to the recently reported literature.[3]

1) a) Moss, T. A.; Fenwick, D. R.; Dixon, D. J. *J. Am. Chem. Soc.* **2008**, *130*, 10076-10077; b) Kim, D. Y.; Park, E. J. *Org. Lett.* **2002**, *4*, 545-547; c) Wang, X.; Lan, Q.; Shirakawa, S.; Maruoka, K. *Chem. Commun.* **2010**, *46*, 321-323.
2) a) Zhdankin, V. V.; Kuehl. C. J.; Krasutsky, A. P.; Bolz, J. T.; Mismash, B.; Woodward, J. K.; Simonsen, A. J. *Tetrahedron Lett.* **1995**, *36*, 7975-7978; b) Akai, S.; Okuno, T.; Egi, M.; Takada, T.; Tohma, H.; Kita, Y. *Heterocycles* **1996**, *42*, 47–51.
3) Wang, Y.-F.; Qiu, J.; Kong, D.; Gao, Y.; Lu, F.; Karmaker, P. G.; Chen, F.-X. *Org. Biomol. Chem.* **2015**, *13*, 365-368.

2. Asymmetric α-Cyanation of β-Ketoesters:

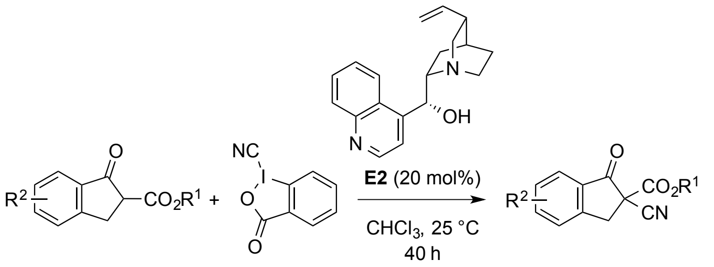

1 **2** (1.2 eq.) **3**

General Procedure: Cyano benziodoxole **2** (1.2 equiv.) was added to a stirred solution of the corresponding β-ketoester **1** and cinchonidine (**E2**, 20 mol%) in chloroform (5 mL per mmol **1**) at room temperature. The reaction mixture was stirred at this temperature for 40 h. The crude product was directly transferred to a silica-gel column and eluted with a gradient of heptane and EtOAc to give the products **3** in the reported yields and enantiopurities (Bromo compound **4** was isolated in 15-20% when using 20 mol% of ammonium bromide catalysts).

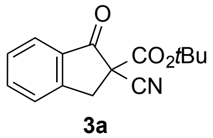

3a
(70%, *e.r.* = 74:26)

Cyanide 3a. Obtained in 70% (0.7 mmol scale) as an oil that crystallizes upon storage in the refrigerator. Analytical data match those reported previously.[3] M.p.: 31-32 °C; $[\alpha]_D^{20}$ (c = 1.2, DCM, *e.r.* = 74:26) = -15; ^{1}H NMR (300 MHz, δ, $CDCl_3$, 298 K): 7.83 (d, *J* = 7.8 Hz, 1H), 7.68-7.73 (m, 1H), 7.44-7.53 (m, 2H), 3.87 (d, *J* = 17.4 Hz, 1H), 3.63 (d, *J* = 17.4 Hz, 1H), 1.48 (s, 9H) ppm; ^{13}C NMR (75 MHz, δ, $CDCl_3$, 298 K): 191.3, 162.8, 151.7, 136.8, 132.3, 128.8, 126.4, 126.2, 116.1, 85.9, 55.2, 37.5, 27.6 (3C) ppm; IR (film): $\tilde{\nu}$ = 2977, 2935, 2245, 1723, 1148, 835, 735 cm^{-1}; HRMS (ESI): *m/z* calcd for $C_{15}H_{15}NO_3$: 280.0960 [M + Na]$^+$; found: 280.0946. Enantiopurity was determined by HPLC (Chiralcel OD-H, eluent: hexane:*i*-PrOH = 90:10, 0.5 mL/min, 10°C, retention times: t_{minor} = 15.7 min, t_{major} = 16.7 min).

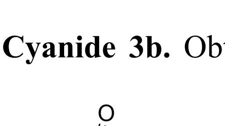

3b
(78%, *e.r.* = 60:40)

Cyanide 3b. Obtained in 78% (0.3 mmol scale) as an oily residue. Analytical data match those reported elsewhere.[3] $[\alpha]_D^{20}$ (c = 1.6, DCM, *e.r.* = 60:40) = -9; ^{1}H NMR (300 MHz, δ, $CDCl_3$, 298 K): 7.84 (d, *J* = 7.7 Hz, 1H), 7.71-7.76 (m, 1H), 7.47-7.56 (m, 2H), 3.95 (d, *J* = 17.1 Hz, 1H), 3.88 (s, 3H), 3.69 (d, *J* = 17.1 Hz, 1H) ppm; ^{13}C NMR (75 MHz, δ, $CDCl_3$, 298 K): 190.6, 164.6, 151.5, 137.0, 132.0, 129.0, 126.5, 126.3, 115.7, 54.7, 54.2, 37.5 ppm; IR (film): $\tilde{\nu}$ = 2958, 2248, 1728, 1238, 906, 749 cm^{-1}; HRMS (ESI): *m/z* calcd for $C_{12}H_9NO_3$: 238.0486 [M + Na]$^+$; found: 238.0478. Enantiopurity was determined by HPLC (Chiralcel OD-H, eluent: hexane:*i*-PrOH = 90:10, 0.5 mL/min, 10°C, t_{minor} = 54.7 min, t_{major} = 56.8 min).

Cyanide 3c. Obtained in 69% (0.2 mmol scale) as an oily residue; $[\alpha]_D^{20}$ (c = 0.3, DCM, *e.r.* = 67:33) = -16; ^{1}H NMR (700 MHz, δ, $CDCl_3$, 298 K): 7.85 (d, *J* = 7.5 Hz, 1H), 7.70-7.75 (m, 1H), 7.46-7.54 (m, 2H), 7.33-7.38 (m, 5H), 5.30 (d, *J* = 12.6 Hz, 1H), 5.26 (d, *J* = 12.6 Hz, 1H), 3.93 (d, *J* = 16.8 Hz, 1H), 3.69 (d, *J* = 16.8 Hz, 1H) ppm; ^{13}C NMR (176 MHz, δ, $CDCl_3$, 298 K): 190.4, 163.9, 151.4, 137.0, 134.2, 132.1, 129.0, 128.72, 128.7, 128.0, 126.5, 126.4, 115.7, 69.3, 54.3, 37.5 ppm; IR (film): $\bar{\nu}$ = 2968, 2244, 1726, 1598, 1371, 1254, 1015, 908, 746 cm^{-1}; HRMS (ESI): *m/z* calcd for $C_{18}H_{13}NO_3$: 314.0796 $[M + Na]^+$; found: 314.0789. The enantioselectivity was determined by HPLC (Chiralcel OD-H, eluent: hexane:*i*-PrOH = 90:10, 0.5 mL/min, 10°C, retention times: t_{minor} = 27.9 min, t_{major} = 29.6 min).

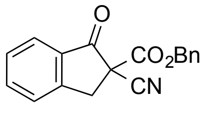

3c
(69%, *e.r.* = 67:33)

Cyanide 3d. Obtained in 80% (0.4 mmol scale) as a white solid. Analytical data match those reported elsewhere.[3] M.p.: 47-50 °C; $[\alpha]_D^{20}$ (c = 0.3, DCM, *e.r.* = 81:19) = -16; ^{1}H NMR (300 MHz, δ, $CDCl_3$, 298 K): 7.83 (d, *J* = 7.8 Hz, 1H), 7.68-7.73 (m, 1H), 7.44-7.53 (m, 2H), 3.87 (d, *J* = 17.4 Hz, 1H), 3.63 (d, *J* = 17.4 Hz, 1H), 2.16 (bs, 3H), 2.11 (bs, 6H), 1.62 (bs, 6H) ppm; ^{13}C NMR (75 MHz, δ, $CDCl_3$, 298 K): 191.3, 162.3, 151.6, 136.7, 132.3, 128.8, 126.4, 126.1, 116.1, 85.8, 55.3, 40.8 (3C), 37.5, 35.8 (3C), 30.9 (3C) ppm; IR (film): $\bar{\nu}$ = 2910, 2246, 1724, 1598, 835, 725 cm^{-1}; HRMS (ESI): *m/z* calcd for $C_{21}H_{21}NO_3$: 358.1426 $[M + Na]^+$; found: 358.1415. The enantioselectivity was determined by HPLC (Chiralcel OD-H, eluent: hexane:*i*-PrOH = 90:10, 0.5 mL/min, 10°C, retention times: t_{major} = 21.0 min, t_{minor} = 22.4 min).

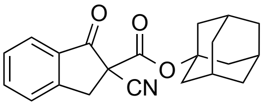

3d
(80%, *e.r.* = 76:24;
81:19 after recryst.)

Cyanide 3e. Obtained in 77% (0.2 mmol scale) as an oily residue. $[\alpha]_D^{20}$ (c = 0.7, DCM, *e.r.* = 75:25) = -18; ^{1}H NMR (300 MHz, δ, $CDCl_3$, 298 K): 7.66 (d, *J* = 7.5 Hz, 1H), 7.51 (d, *J* = 7.5 Hz, 1H), 7.38 (t, *J* = 7.5 Hz, 1H), 3.77 (d, *J* = 17.1 Hz, 1H), 3.51 (d, *J* = 17.1 Hz, 1H), 2.38 (s, 3H), 1.50 (s, 9H); ^{13}C NMR (75 MHz, δ, $CDCl_3$, 298 K): 191.5, 162.9, 150.6, 137.3, 135.9, 132.1, 129.0, 123.6, 116.2, 85.8, 55.1, 36.5, 27.6 (3C), 17.7 ppm; IR (film): $\bar{\nu}$ = 2981, 2935, 2246, 1724, 1591, 1251, 1148, 832 cm^{-1}; HRMS (ESI): *m/z* calcd for $C_{16}H_{17}NO_3$: 294.1107 $[M + Na]^+$; found: 294.1104. The enantioselectivity was determined by HPLC (Chiralcel OD-H, eluent: hexane:*i*-PrOH = 90:10, 0.5 mL/min, 10°C, retention times: t_{minor} = 15.2 min, t_{major} = 17.6 min).

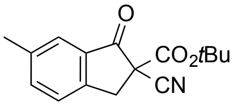

3e
(77%, *e.r.* = 75:25)

Cyanide 3f. Obtained in 78% (0.3 mmol scale) as an oily residue. $[\alpha]_D^{20}$ (c = 1.3, DCM, *e.r.* = 75:25) = -9; ^{1}H NMR (300 MHz, δ, $CDCl_3$, 298 K): 7.81-7.82 (m, 1H), 7.76 (dd, *J* = 1.8, 8.1 Hz, 1H), 7.44-7.46 (m, 1H), 3.83 (d, *J* = 17.1 Hz, 1H), 3.59 (d, *J* = 17.1 Hz, 1H), 1.50 (s, 9H), 1.34 (s, 9H) ppm; ^{13}C NMR (75 MHz, δ, $CDCl_3$, 298 K): 191.5, 163.0, 152.5, 149.2, 134.8, 132.2, 126.0, 122.3, 116.3, 85.7, 55.5, 37.1, 34.9, 31.2 (3C), 27.6 (3C) ppm; IR (film): $\bar{\nu}$ = 2982, 2934, 2245, 1725, 1591, 1250, 1147, 831 cm^{-1}; HRMS (ESI): *m/z* calcd for $C_{19}H_{23}NO_3$: 336.1580 $[M + Na]^+$; found: 336.1570. The enantioselectivity was determined by HPLC (Chiralcel OD-H, eluent: hexane:*i*-PrOH = 90:10, 0.5 mL/min, 10°C, retention times: t_{minor} = 10.4 min, t_{major} = 12.4 min).

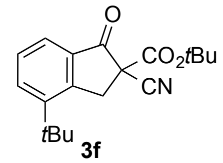

3f
(78%, *e.r.* = 75:25)

Cyanide 3g. Obtained in 86% (0.3 mmol scale) as an oily residue. $[\alpha]_D^{20}$ (c = 1.2, DCM, *e.r.* = 73:27) = -12; ^{1}H NMR (300 MHz, δ, $CDCl_3$, 298 K): 7.76 (d, *J* = 8.1 Hz, 1H), 7.52-7.54 (m, 1H), 7.43-7.47 (m, 1H), 3.85 (d, *J* = 17.4 Hz, 1H), 3.60 (d, *J* = 17.4 Hz, 1H), 1.49 (s, 9H) ppm; ^{13}C NMR (75 MHz, δ, $CDCl_3$, 298 K): 189.8, 162.5, 153.0, 143.7, 130.7, 129.7, 127.1, 126.7, 115.7, 86.2, 55.2, 37.0, 27.6 (3C) ppm; IR (film): $\bar{\nu}$ = 2980, 2245, 1723, 1598, 1371, 1253, 1219, 1016, 907, 886 cm^{-1}; HRMS (ESI): *m/z* calcd for $C_{15}H_{14}ClNO_3$: 314.0570 $[M + Na]^+$; found: 314.0559. The enantioselectivity was determined by HPLC (Chiralpak AD-H, eluent: hexane:*i*-PrOH = 90:10, 0.8 mL/min, 10°C, retention times: t_{minor} = 12.2 min, t_{major} = 13.3 min).

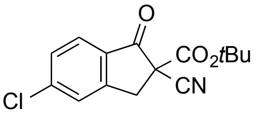

3g
(86%, *e.r.* = 73:27)

Cyanide 3h. Obtained in 81% (0.2 mmol scale) as an oily residue. $[\alpha]_D^{20}$ (c = 0.4, DCM, *e.r.* = 73:27) = -11; ^{1}H NMR (300 MHz, δ, $CDCl_3$, 298 K): 7.76 (d, *J* = 8.1 Hz, 1H), 7.53 (s, 1H), 7.43-7.47 (m, 1H), 3.85 (d, *J* = 17.4 Hz, 1H), 3.60 (d, *J* = 17.4 Hz, 1H), 1.49 (s, 9H) ppm; ^{13}C NMR (75 MHz, δ, $CDCl_3$, 298 K): 190.0, 162.4, 153.0, 132.7, 132.6, 131.1, 129.8, 127.1, 115.7, 86.2, 55.2, 37.0, 27.6 (3C) ppm; IR (film): $\bar{\nu}$ = 2978, 2246, 1725, 1598, 1371, 1252, 1219, 1016, 865, 840 cm^{-1}; HRMS (ESI): *m/z* calcd for $C_{15}H_{14}BrNO_3$: 358.0065 $[M + Na]^+$; found: 358.0050. The enantioselectivity was determined by HPLC (Chiralpak AD-H, eluent: hexane:*i*-PrOH = 90:10, 0.8 mL/min, 10°C, retention times: t_{minor} = 11.7 min, t_{major} = 12.7 min).

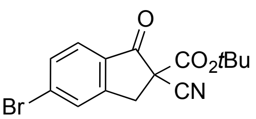

3h
(81%, *e.r.* = 73:27)

Bromide 4. Isolated in 15-20% when using 20 mol% of ammonium bromide catalysts.

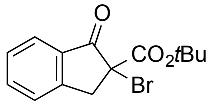

4

Analytical data are in accordance to those reported in literature.[4] ^{1}H NMR (300 MHz, δ, $CDCl_3$, 298 K): 7.79 (d, *J* = 7.8 Hz, 1H), 7.67-7.57 (m, 1H), 7.43-7.33 (m, 2H), 4.05 (d, *J* = 18.0 Hz, 1H), 3.58 (d, *J* = 18.0 Hz, 1H), 1.39 (s, 9H) ppm; ^{13}C NMR (75 MHz, δ, $CDCl_3$, 298 K): 195.6, 165.8, 150.3, 136.1, 132.5, 128.4, 126.2, 125.9, 84.5, 59.9, 44.0, 27.7 (3C) ppm; HRMS (ESI): *m/z* calcd for $C_{14}H_{15}BrO_3$: 328.0543 $[M + NH_4]^+$; found: 328.0542.

4) Liu, G.; Wang, X.; Lu, X.; Xu, X.-H.; Tokunaga, E.; Shibata, N. *ChemistryOpen* **2012**, *1*, 227-231.

3. Copies of NMR spectra:

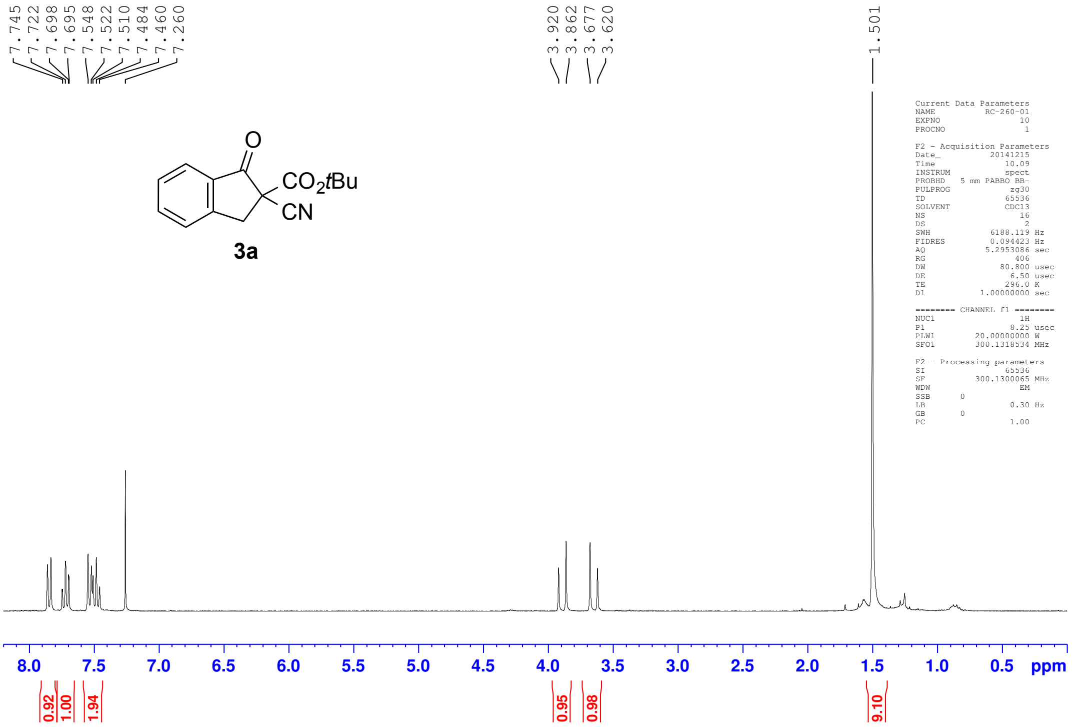

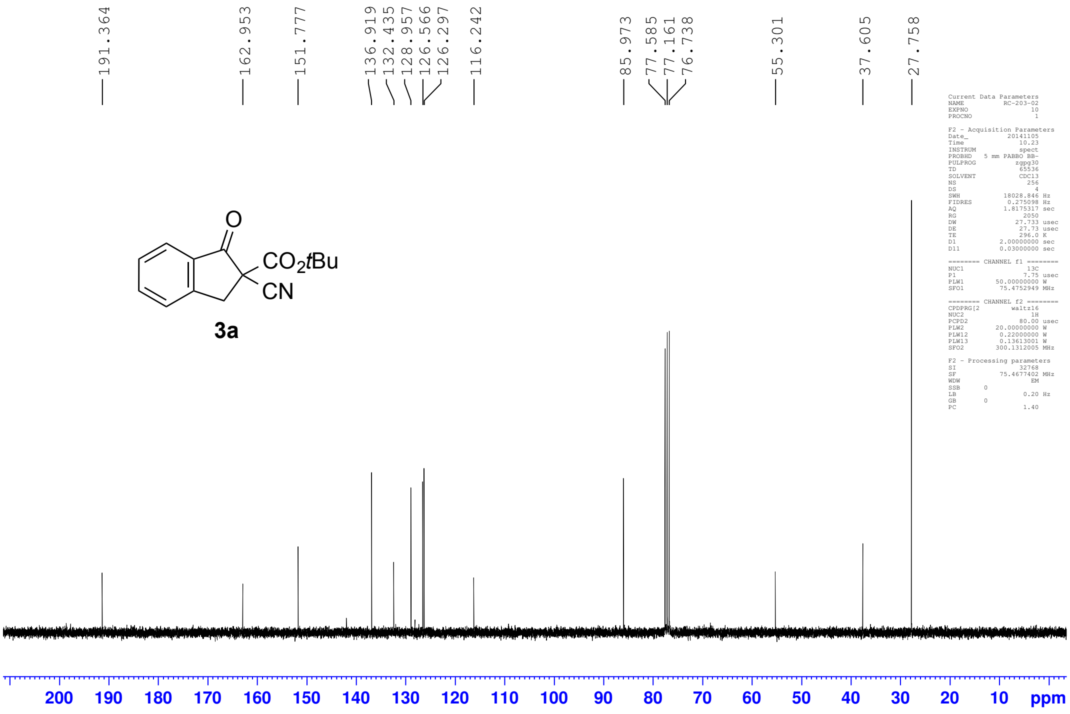

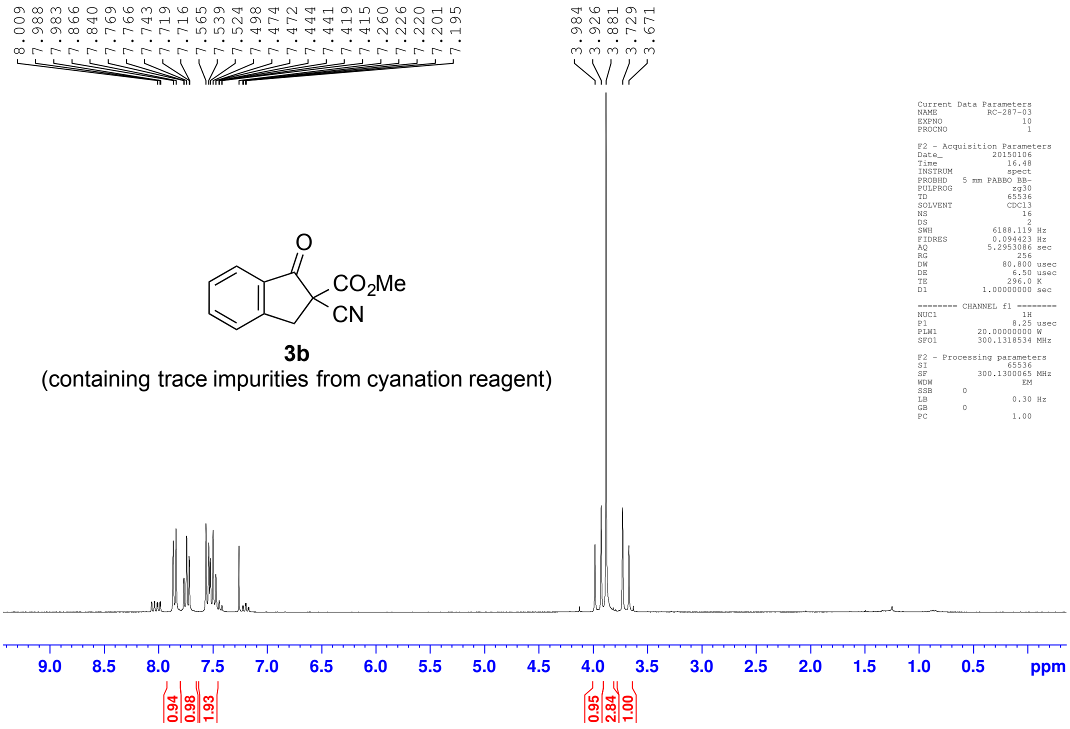
8.009 7.988 7.983 7.866 7.840 7.769 7.766 7.743 7.719 7.716 7.565 7.539 7.524 7.498 7.474 7.472 7.444 7.441 7.419 7.415 7.260 7.226 7.220 7.201 7.195
3.984 3.926 3.881 3.729 3.671
O
CO_2Me
CN
3b
(containing trace impurities from cyanation reagent)
0.94 0.98 1.93
0.95 2.84 1.00
9.0 8.5 8.0 7.5 7.0 6.5 6.0 5.5 5.0 4.5 4.0 3.5 3.0 2.5 2.0 1.5 1.0 0.5 ppm

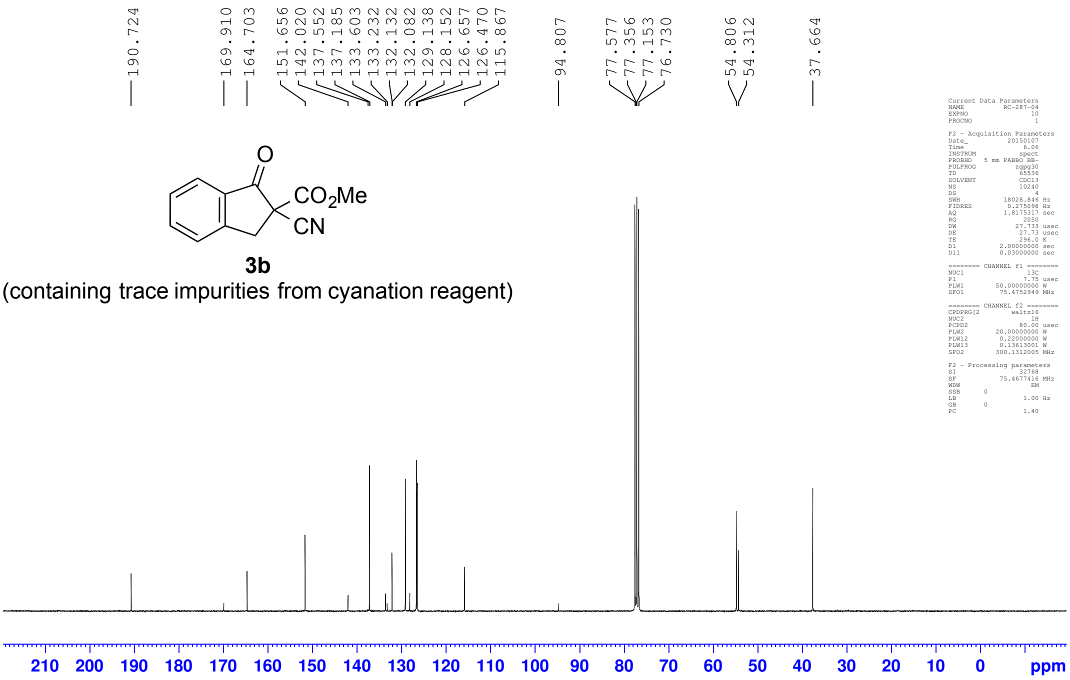
190.724
169.910 164.703
151.656 142.020 137.552 137.185 133.603 133.232 132.132 132.082 129.138 128.152 126.657 126.470 115.867
94.807
77.577 77.356 77.153 76.730
54.806 54.312
37.664
O
CO_2Me
CN
3b
(containing trace impurities from cyanation reagent)
210 200 190 180 170 160 150 140 130 120 110 100 90 80 70 60 50 40 30 20 10 0 ppm

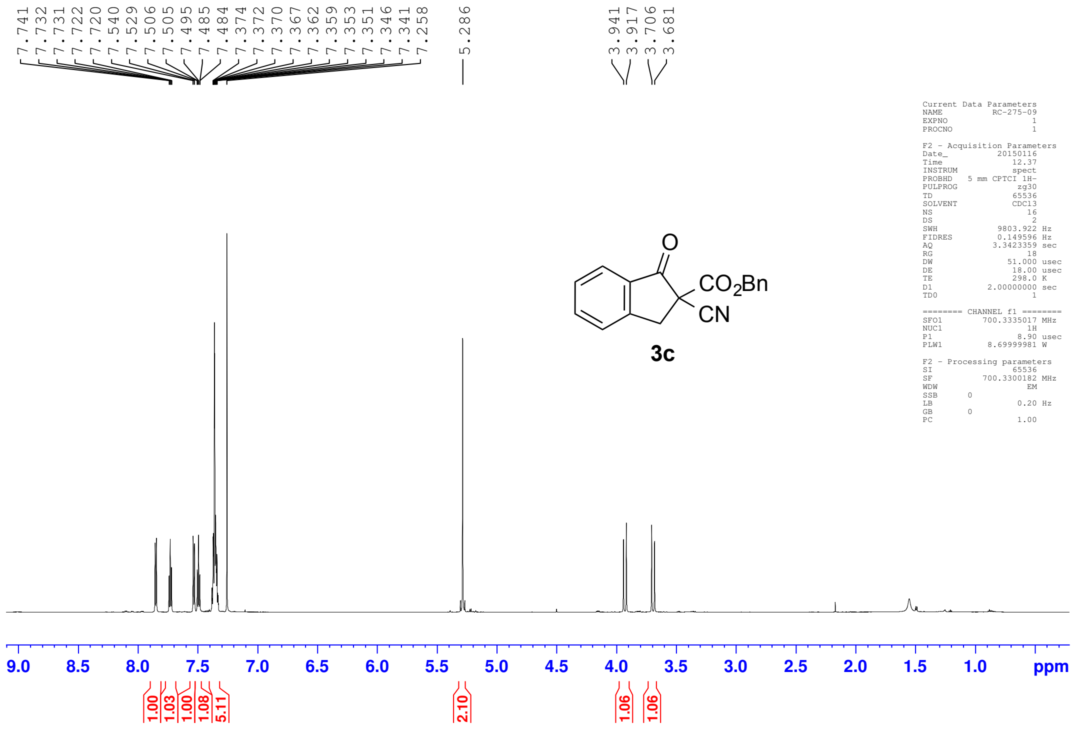
7.741
7.732
7.731
7.722
7.720
7.540
7.529
7.506
7.505
7.495
7.485
7.484
7.374
7.372
7.370
7.367
7.362
7.359
7.353
7.351
7.346
7.341
7.258
5.286
3.941
3.917
3.706
3.681
O
CO_2Bn
CN
3c
9.0 8.5 8.0 7.5 7.0 6.5 6.0 5.5 5.0 4.5 4.0 3.5 3.0 2.5 2.0 1.5 1.0 ppm
1.00 1.03 1.00 1.08 5.11 2.10 1.06 1.06

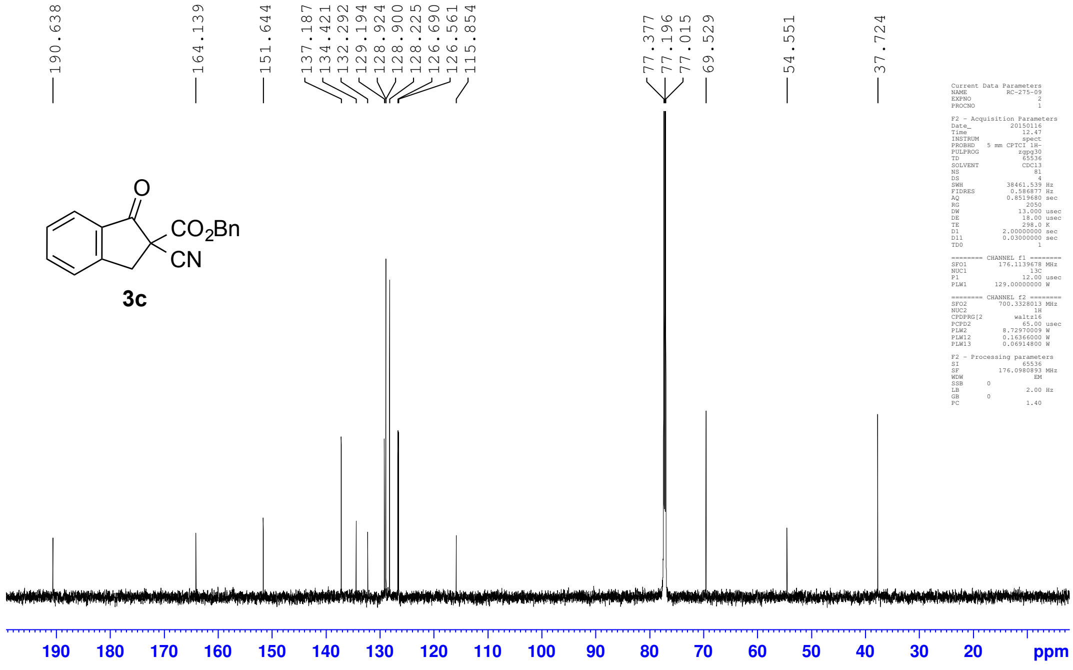
190.638
164.139
151.644
137.187
134.421
132.292
129.194
128.924
128.900
128.225
126.690
126.561
115.854
77.377
77.196
77.015
69.529
54.551
37.724
O
CO_2Bn
CN
3c
190 180 170 160 150 140 130 120 110 100 90 80 70 60 50 40 30 20 ppm

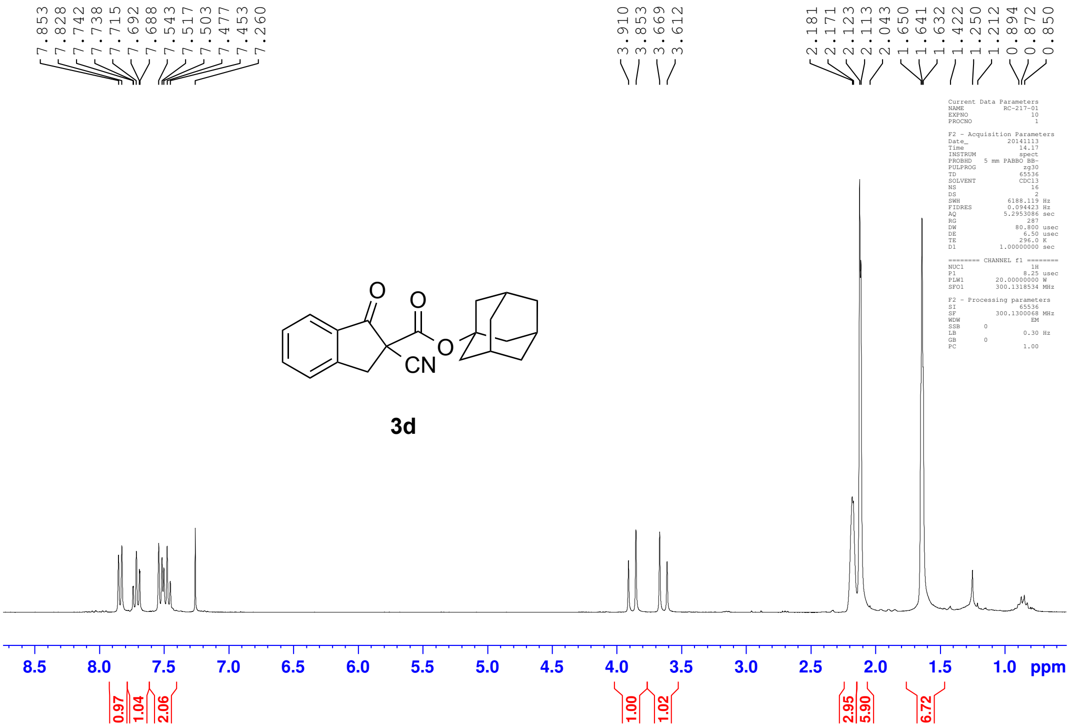
7.853 7.828 7.742 7.738 7.715 7.692 7.688 7.543 7.517 7.503 7.477 7.453 7.260
3.910 3.853 3.669 3.612
2.181 2.171 2.123 2.113 2.043 1.650 1.641 1.632 1.422 1.250 1.212 0.894 0.872 0.850
O
O
O
CN
3d
8.5 8.0 7.5 7.0 6.5 6.0 5.5 5.0 4.5 4.0 3.5 3.0 2.5 2.0 1.5 1.0 ppm
0.97 1.04 2.06 1.00 1.02 2.95 5.90 6.72

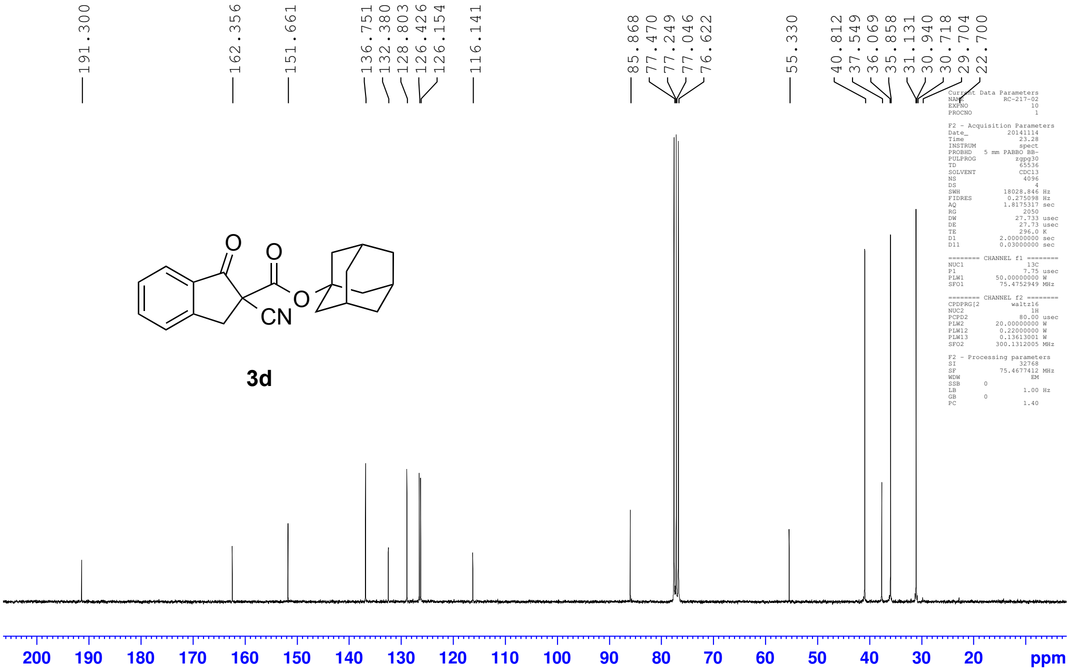
191.300
162.356
151.661
136.751 132.380 128.803 126.426 126.154
116.141
85.868 77.470 77.249 77.046 76.622
55.330
40.812 37.549 36.069 35.858 31.131 30.940 30.718 29.704 22.700
O
O
O
CN
3d
200 190 180 170 160 150 140 130 120 110 100 90 80 70 60 50 40 30 20 ppm

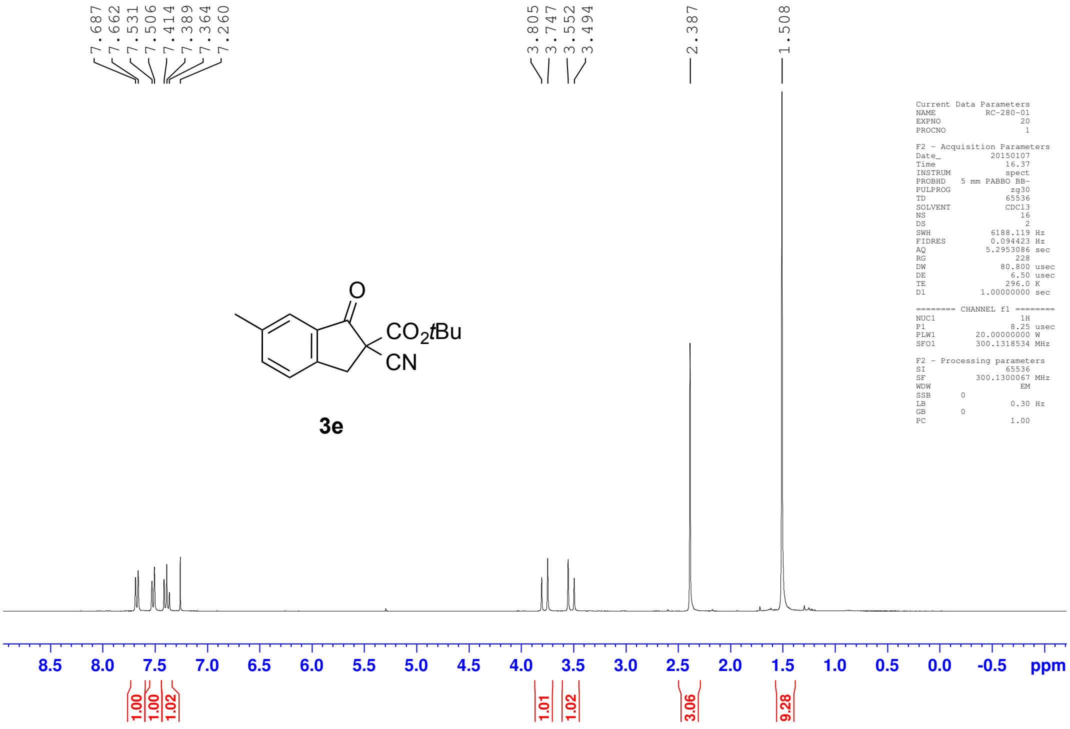
7.687
7.662
7.531
7.506
7.414
7.389
7.364
7.260
3.805
3.747
3.552
3.494
2.387
1.508
O
CO2tBu
CN
3e
8.5 8.0 7.5 7.0 6.5 6.0 5.5 5.0 4.5 4.0 3.5 3.0 2.5 2.0 1.5 1.0 0.5 0.0 -0.5 ppm
1.00 1.00 1.02 1.01 1.02 3.06 9.28

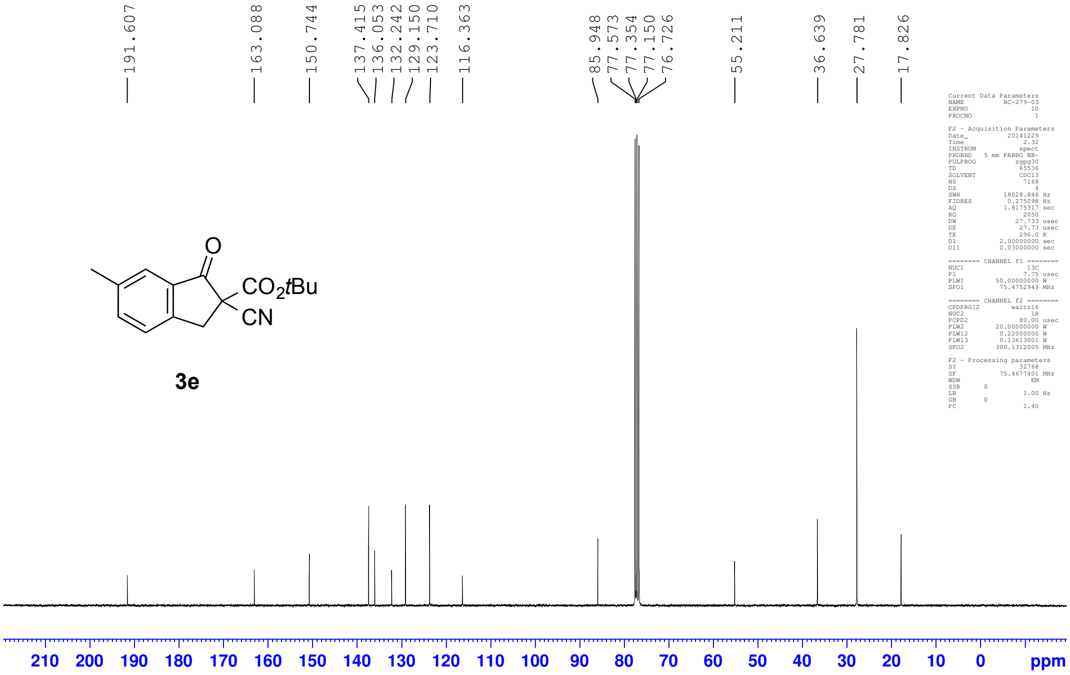
191.607
163.088
150.744
137.415
136.053
132.242
129.150
123.710
116.363
85.948
77.573
77.354
77.150
76.726
55.211
36.639
27.781
17.826
O
CO2tBu
CN
3e
210 200 190 180 170 160 150 140 130 120 110 100 90 80 70 60 50 40 30 20 10 0 ppm

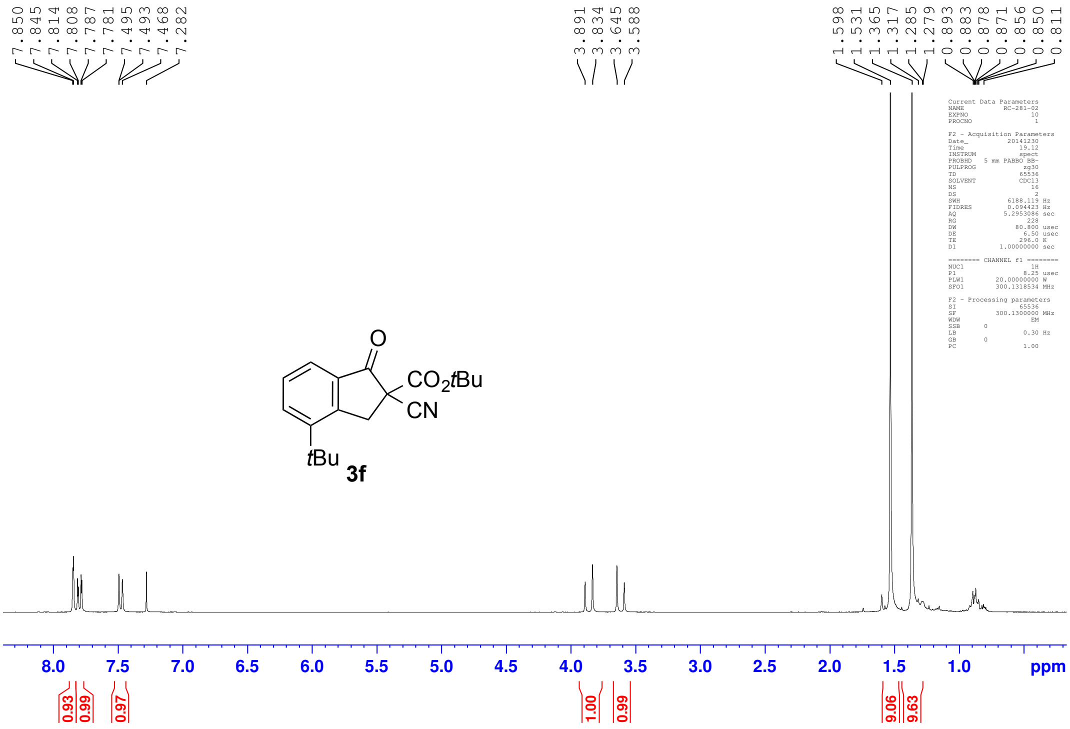
7.850
7.845
7.814
7.808
7.787
7.781
7.495
7.493
7.468
7.282
3.891
3.834
3.645
3.588
1.598
1.531
1.365
1.317
1.285
1.279
0.893
0.883
0.878
0.871
0.856
0.850
0.811
O
CO2tBu
CN
tBu
3f
8.0 7.5 7.0 6.5 6.0 5.5 5.0 4.5 4.0 3.5 3.0 2.5 2.0 1.5 1.0 ppm
0.93
0.99
0.97
1.00
0.99
9.06
9.63

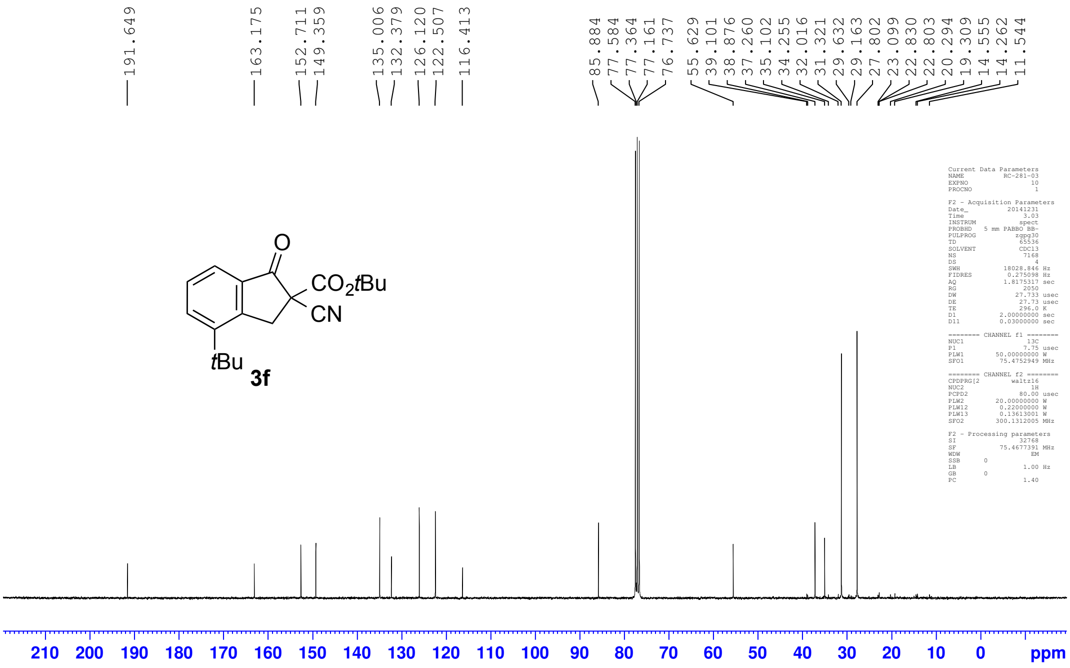
191.649
163.175
152.711
149.359
135.006
132.379
126.120
122.507
116.413
85.884
77.584
77.364
77.161
76.737
55.629
39.101
38.876
37.260
35.102
34.255
32.016
31.321
29.632
29.163
27.802
23.099
22.830
22.803
20.294
19.309
14.555
14.262
11.544
O
CO2tBu
CN
tBu
3f
210 200 190 180 170 160 150 140 130 120 110 100 90 80 70 60 50 40 30 20 10 0 ppm

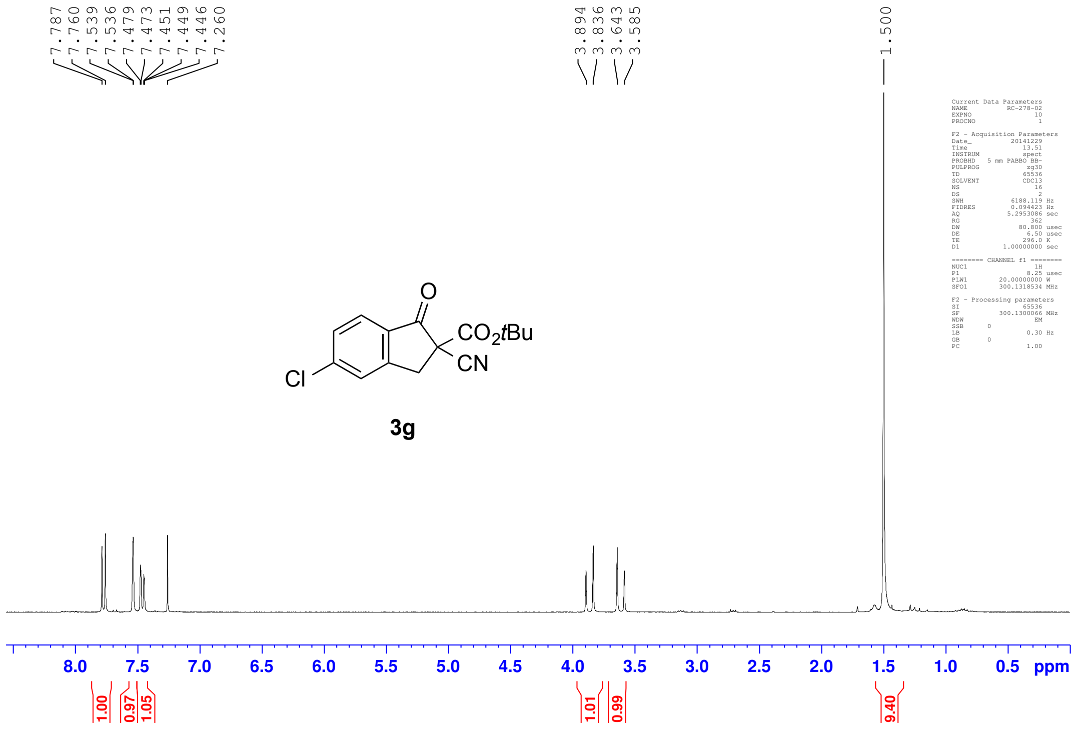
7.787
7.760
7.539
7.536
7.479
7.473
7.451
7.449
7.446
7.260
3.894
3.836
3.643
3.585
1.500
CO2tBu
CN
Cl
O
3g
8.0 7.5 7.0 6.5 6.0 5.5 5.0 4.5 4.0 3.5 3.0 2.5 2.0 1.5 1.0 0.5 ppm
1.00
0.97
1.05
1.01
0.99
9.40

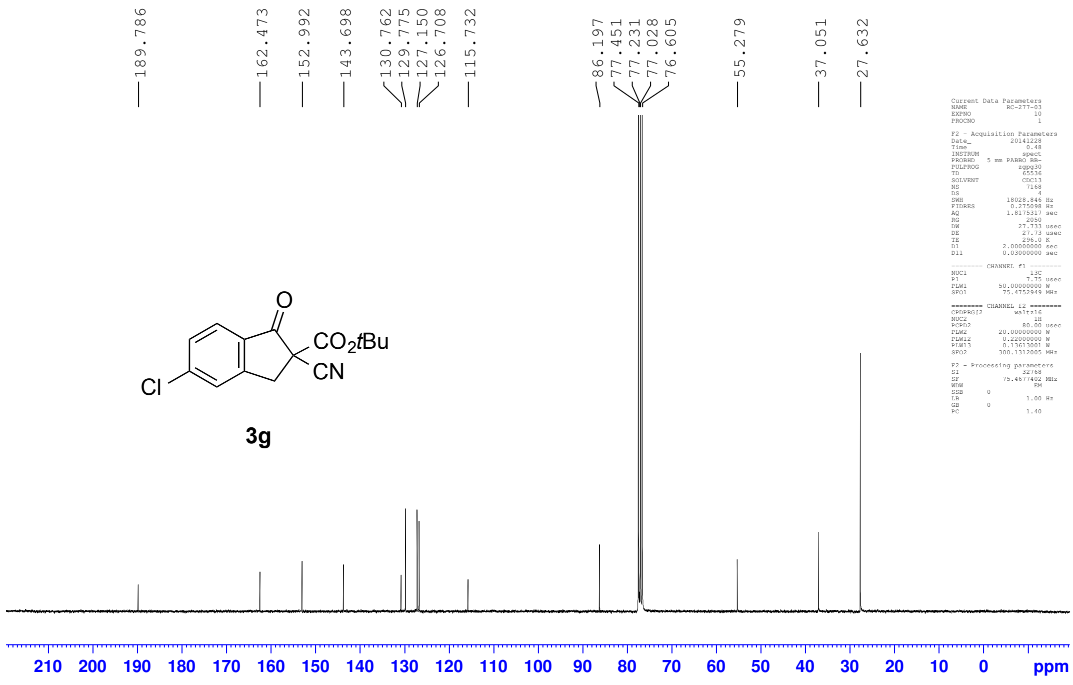
189.786
162.473
152.992
143.698
130.762
129.775
127.150
126.708
115.732
86.197
77.451
77.231
77.028
76.605
55.279
37.051
27.632
CO2tBu
CN
Cl
O
3g
210 200 190 180 170 160 150 140 130 120 110 100 90 80 70 60 50 40 30 20 10 0 ppm

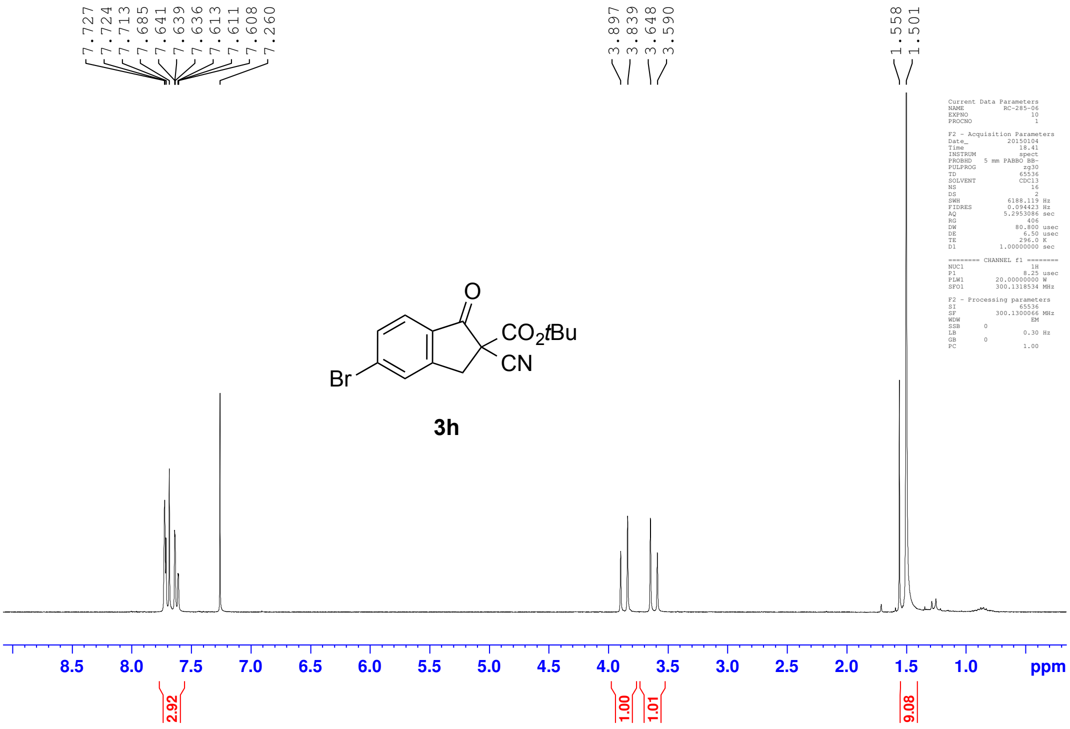

7.727 7.724 7.713 7.685 7.641 7.639 7.636 7.613 7.611 7.608 7.260
3.897 3.839 3.648 3.590
1.558 1.501
O
CO2tBu
CN
Br
3h
8.5 8.0 7.5 7.0 6.5 6.0 5.5 5.0 4.5 4.0 3.5 3.0 2.5 2.0 1.5 1.0 ppm
2.92
1.00
1.01
9.08

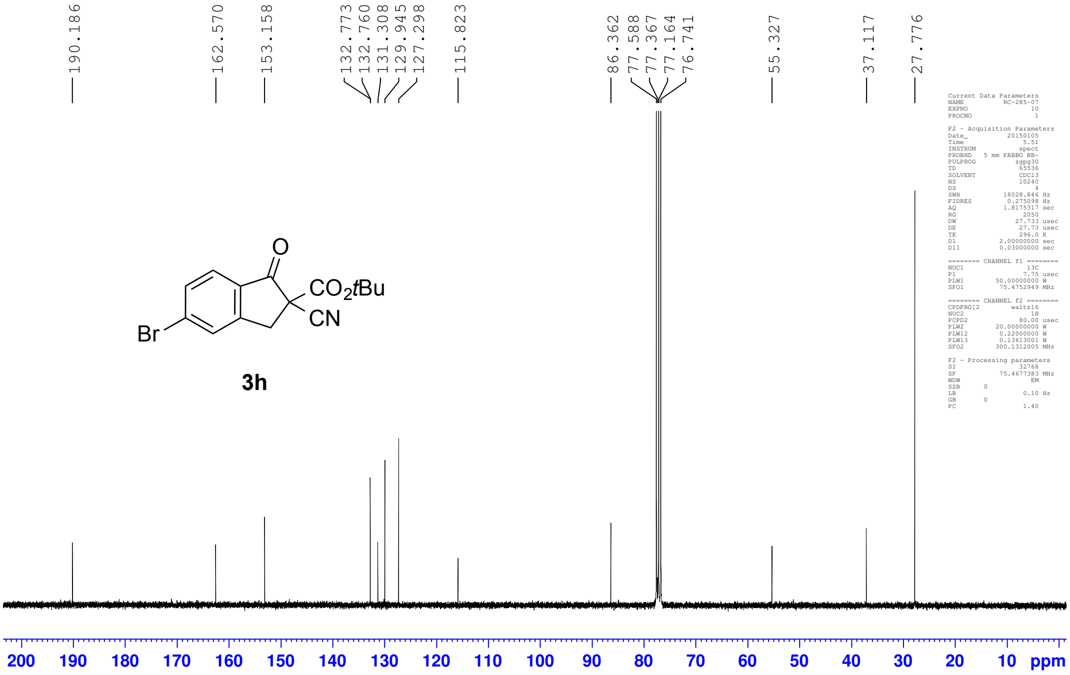

190.186
162.570
153.158
132.773 132.760 131.308 129.945 127.298
115.823
86.362 77.588 77.367 77.164 76.741
55.327
37.117
27.776
O
CO2tBu
CN
Br
3h
200 190 180 170 160 150 140 130 120 110 100 90 80 70 60 50 40 30 20 10 ppm

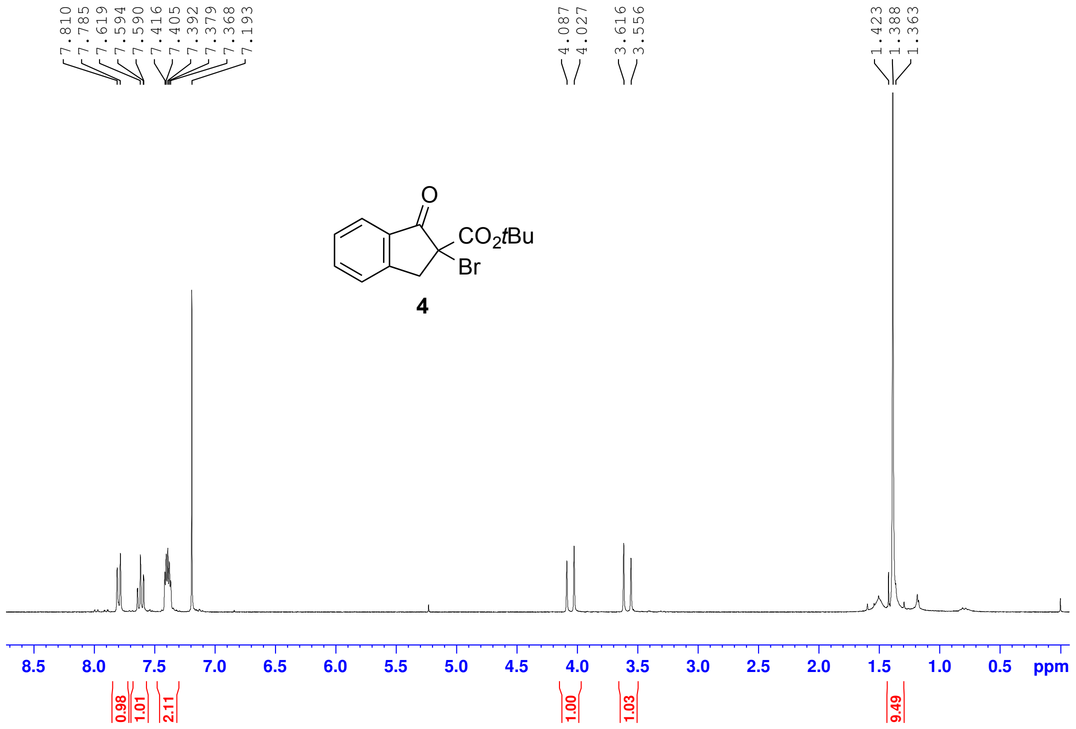
7.810
7.785
7.619
7.594
7.590
7.416
7.405
7.392
7.379
7.368
7.193
4.087
4.027
3.616
3.556
1.423
1.388
1.363
O
CO2tBu
Br
4
8.5
8.0
7.5
7.0
6.5
6.0
5.5
5.0
4.5
4.0
3.5
3.0
2.5
2.0
1.5
1.0
0.5
ppm
0.98
1.01
2.11
1.00
1.03
9.49

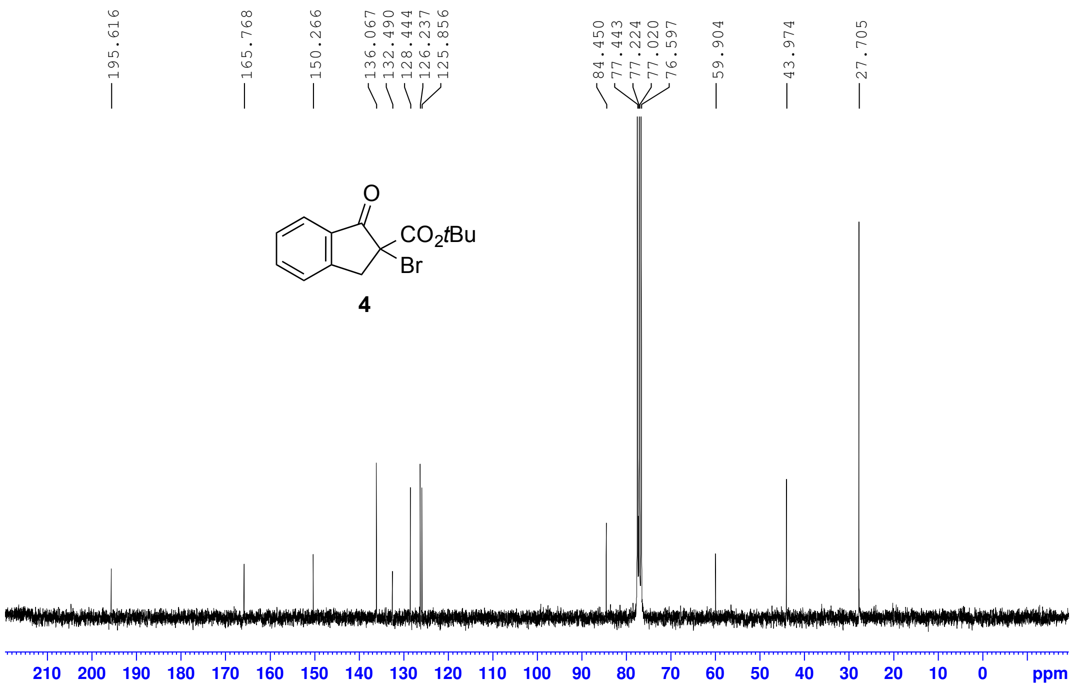
195.616
165.768
150.266
136.067
132.490
128.444
126.237
125.856
84.450
77.443
77.224
77.020
76.597
59.904
43.974
27.705
O
CO2tBu
Br
4
210
200
190
180
170
160
150
140
130
120
110
100
90
80
70
60
50
40
30
20
10
0
ppm

4. HPLC Chromatograms:

Operator:Admin Timebase:Summit_1 Sequence:WAS_20141107_RC_ODH

Page 1-2
6.1.2015 3:47 PM

2 RC-203

Sample Name:	**RC-203**	*Injection Volume:*	**10,0**
Vial Number:	**RA1**	*Channel:*	**UV_VIS_2**
Sample Type:	**unknown**	*Wavelength:*	**250**
Control Program:	**OD_H_90Min_9_1_flow05**	*Bandwidth:*	**n.a.**
Quantif. Method:	**OD_H**	*Temperature/Column:*	**10**
Recording Time:	**7.11.2014 12:16**	*Flow ml/min:*	**0,500**
Run Time (min):	**35,75**	*Sample Amount:*	**1,0000**

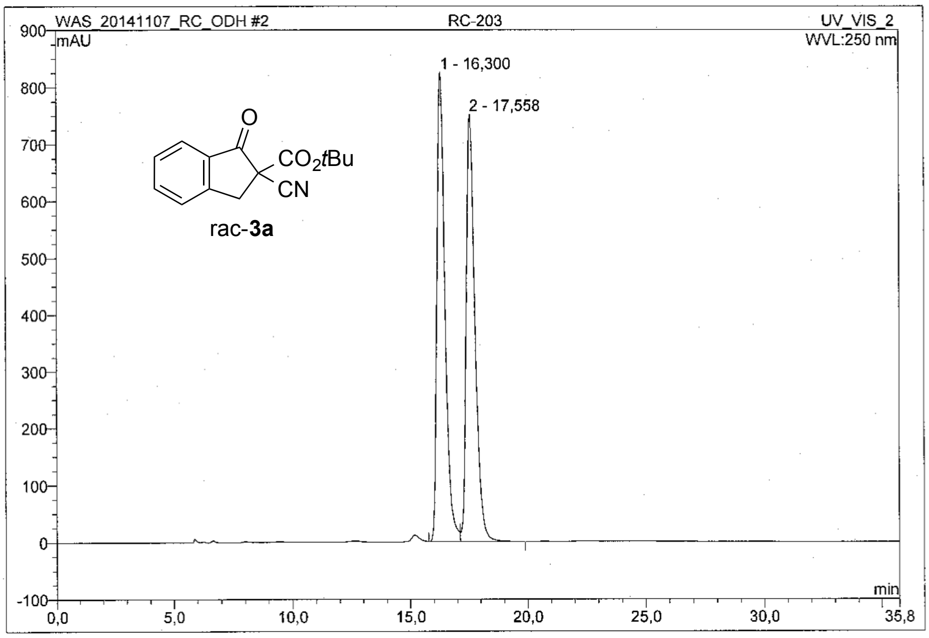

No.	Ret.Time min	Peak Name	Height mAU	Area mAU*min	Rel.Area %	Amount	Type
1	16,30	n.a.	824,801	326,891	49,71	n.a.	BM
2	17,56	n.a.	751,079	330,686	50,29	n.a.	MB
**Total:**			1575,880	657,578	100,00	0,000	

default/Integration

Chromeleon (c) Dionex 1996-2006
Version 6.80 SR12 Build 3578 (207169)

3 RC-255

Sample Name:	**RC-255**	*Injection Volume:*	**10,0**
Vial Number:	**RA3**	*Channel:*	**UV_VIS_2**
Sample Type:	**unknown**	*Wavelength:*	**250**
Control Program:	**OD_H_30Min_9_1_flow05**	*Bandwidth:*	**n.a.**
Quantif. Method:	**OD_H**	*Temperature/Column:*	**10**
Recording Time:	**11.12.2014 14:51**	*Flow ml/min:*	**0,500**
Run Time (min):	**30,00**	*Sample Amount:*	**1,0000**

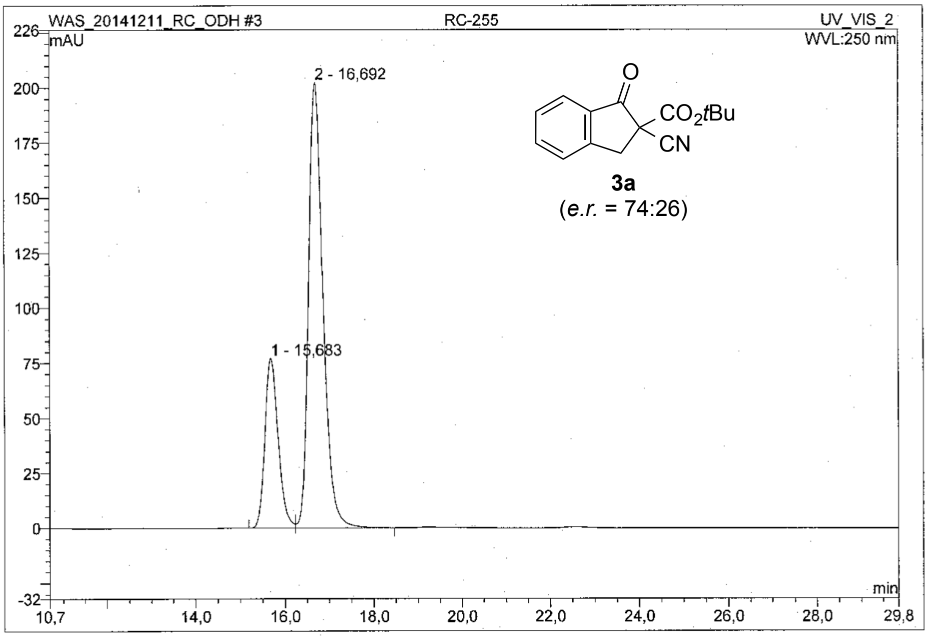

No.	Ret.Time min	Peak Name	Height mAU	Area mAU*min	Rel.Area %	Amount	Type
1	15,68	n.a.	77,155	26,822	25,80	n.a.	BM
2	16,69	n.a.	202,327	77,141	74,20	n.a.	MB
**Total:**			279,482	103,963	100,00	0,000	

4 RC-264

Sample Name:	**RC-264**	*Injection Volume:*	**10,0**
Vial Number:	**RA4**	*Channel:*	**UV_VIS_2**
Sample Type:	**unknown**	*Wavelength:*	**250**
Control Program:	**OD_H_60Min_7_3_flow05**	*Bandwidth:*	**n.a.**
Quantif. Method:	**OD_H**	*Temperature/Column:*	**10**
Recording Time:	**18.12.2014 14:38**	*Flow ml/min:*	**0,500**
Run Time (min):	**60,00**	*Sample Amount:*	**1,0000**

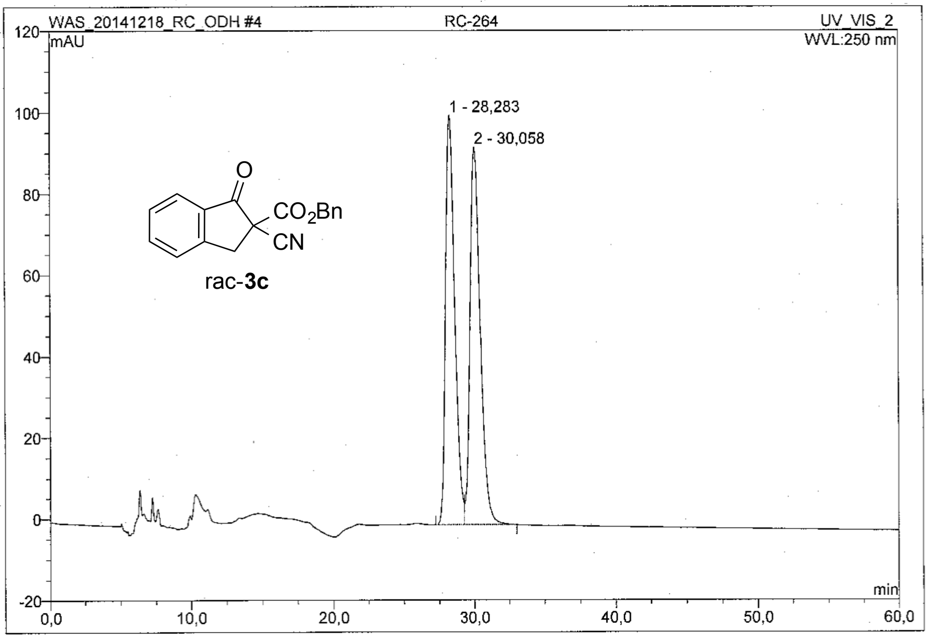

No.	Ret.Time min	Peak Name	Height mAU	Area mAU*min	Rel.Area %	Amount	Type
1	28,28	n.a.	100,853	76,333	49,29	n.a.	BM
2	30,06	n.a.	92,980	78,531	50,71	n.a.	MB
**Total:**			193,833	154,865	100,00	0,000	

2 RC-275-1

Sample Name:	**RC-275-1**	*Injection Volume:*	**10,0**
Vial Number:	**RA2**	*Channel:*	**UV_VIS_2**
Sample Type:	**unknown**	*Wavelength:*	**250**
Control Program:	**OD_H_60Min_7_3_flow05**	*Bandwidth:*	**n.a.**
Quantif. Method:	**OD_H**	*Temperature/Column:*	**10**
Recording Time:	**25.12.2014 15:33**	*Flow ml/min:*	**0,500**
Run Time (min):	**58,84**	*Sample Amount:*	**1,0000**

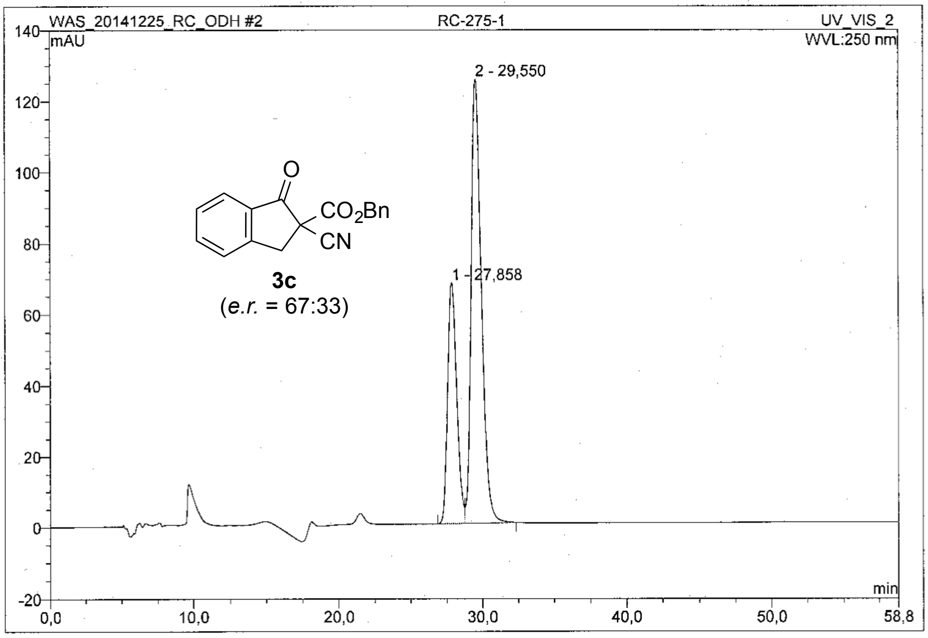

No.	Ret.Time min	Peak Name	Height mAU	Area mAU*min	Rel.Area %	Amount	Type
1	27,86	n.a.	67,809	50,011	32,71	n.a.	BM
2	29,55	n.a.	124,991	102,879	67,29	n.a.	MB
**Total:**			192,800	152,890	100,00	0,000	

Chromeleon (c) Dionex 1996-2006
Version 6.80 SR12 Build 3578 (207169)

2 RC-217

Sample Name:	**RC-217**	*Injection Volume:*	**10,0**
Vial Number:	**RA2**	*Channel:*	**UV_VIS_2**
Sample Type:	**unknown**	*Wavelength:*	**250**
Control Program:	**OD_H_60Min_9_1_flow05**	*Bandwidth:*	**n.a.**
Quantif. Method:	**OD_H**	*Temperature/Column:*	**10**
Recording Time:	**13.11.2014 15:05**	*Flow ml/min:*	**0,500**
Run Time (min):	**60,00**	*Sample Amount:*	**1,0000**

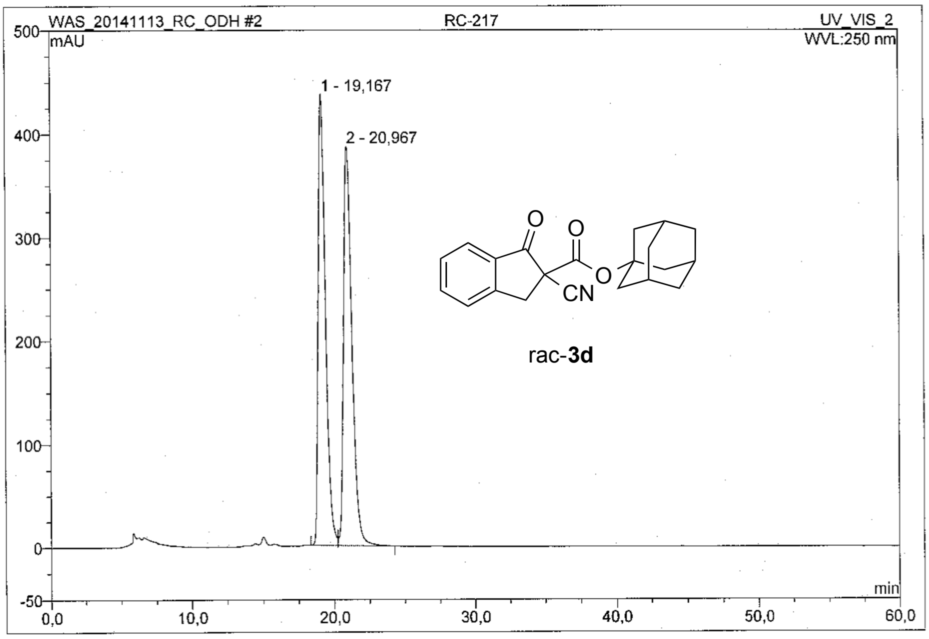

No.	Ret.Time min	Peak Name	Height mAU	Area mAU*min	Rel.Area %	Amount	Type
1	19,17	n.a.	436,767	258,914	49,42	n.a.	BM
2	20,97	n.a.	386,856	265,010	50,58	n.a.	MB
**Total:**			823,623	523,924	100,00	0,000	

Chromeleon (c) Dionex 1996-2006
Version 6.80 SR12 Build 3578 (207169)

2 RC-274

Sample Name:	**RC-274**	*Injection Volume:*	**10,0**
Vial Number:	**RA2**	*Channel:*	**UV_VIS_2**
Sample Type:	**unknown**	*Wavelength:*	**250**
Control Program:	**OD_H_30Min_9_1_flow05**	*Bandwidth:*	**n.a.**
Quantif. Method:	**OD_H**	*Temperature/Column:*	**10**
Recording Time:	**24.12.2014 18:40**	*Flow ml/min:*	**0,500**
Run Time (min):	**30,00**	*Sample Amount:*	**1,0000**

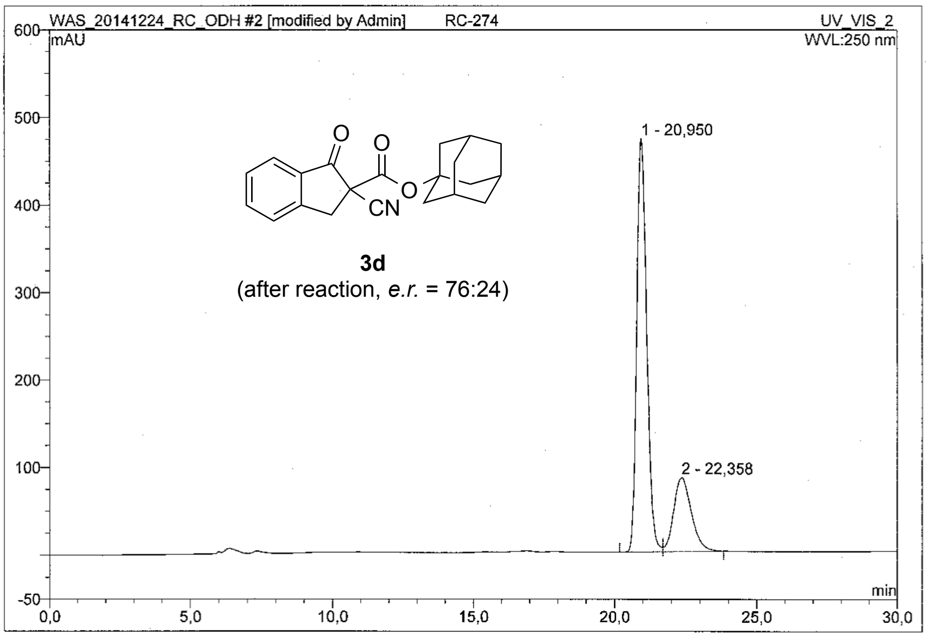

No.	Ret.Time min	Peak Name	Height mAU	Area mAU*min	Rel.Area %	Amount	Type
1	20,95	n.a.	472,337	190,396	75,97	n.a.	BM *
2	22,36	n.a.	84,577	60,226	24,03	n.a.	MB*
**Total:**			556,914	250,622	100,00	0,000	

1 RC-274

Sample Name:	**RC-274**	*Injection Volume:*	**10,0**
Vial Number:	**RA1**	*Channel:*	**UV_VIS_2**
Sample Type:	**unknown**	*Wavelength:*	**250**
Control Program:	**OD_H_30Min_9_1_flow05**	*Bandwidth:*	**n.a.**
Quantif. Method:	**OD_H**	*Temperature/Column:*	**10**
Recording Time:	**30.12.2014 15:17**	*Flow ml/min:*	**0,500**
Run Time (min):	**30,00**	*Sample Amount:*	**1,0000**

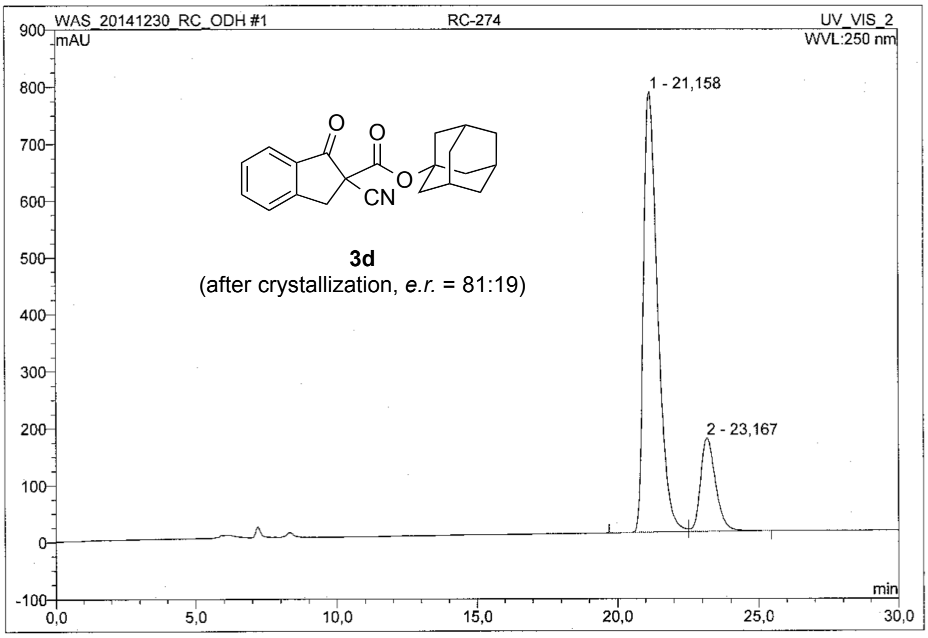

No.	Ret.Time min	Peak Name	Height mAU	Area mAU*min	Rel.Area %	Amount	Type
1	21,16	n.a.	774,331	448,082	81,48	n.a.	BM
2	23,17	n.a.	165,084	101,861	18,52	n.a.	MB
Total:			939,414	549,944	100,00	0,000	

3 RC-279

Sample Name:	**RC-279**	*Injection Volume:*	**10,0**
Vial Number:	**RA3**	*Channel:*	**UV_VIS_1**
Sample Type:	**unknown**	*Wavelength:*	**220**
Control Program:	**OD_H_30Min_9_1_flow05**	*Bandwidth:*	**n.a.**
Quantif. Method:	**OD_H**	*Temperature/Column:*	**10**
Recording Time:	**29.12.2014 16:57**	*Flow ml/min:*	**0,500**
Run Time (min):	**30,00**	*Sample Amount:*	**1,0000**

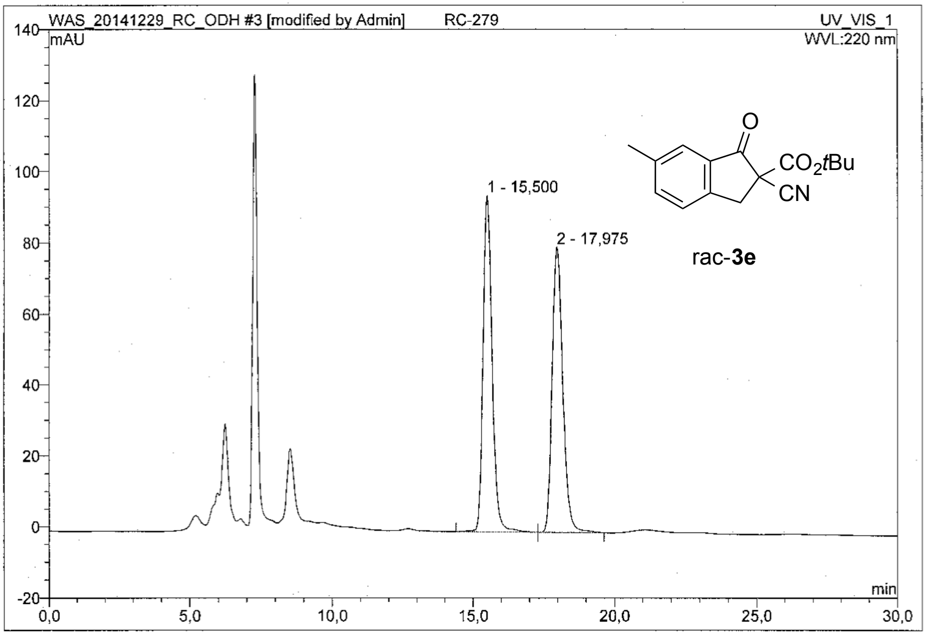

No.	Ret.Time min	Peak Name	Height mAU	Area mAU*min	Rel.Area %	Amount	Type
1	15,50	n.a.	94,666	35,404	50,29	n.a.	BMb
2	17,98	n.a.	80,364	34,999	49,71	n.a.	bMB
**Total:**			175,030	70,403	100,00	0,000	

2 RC-280

Sample Name:	**RC-280**	*Injection Volume:*	**10,0**
Vial Number:	**RA2**	*Channel:*	**UV_VIS_1**
Sample Type:	**unknown**	*Wavelength:*	**220**
Control Program:	**OD_H_30Min_9_1_flow05**	*Bandwidth:*	**n.a.**
Quantif. Method:	**OD_H**	*Temperature/Column:*	**10**
Recording Time:	**31.12.2014 16:00**	*Flow ml/min:*	**0,500**
Run Time (min):	**30,00**	*Sample Amount:*	**1,0000**

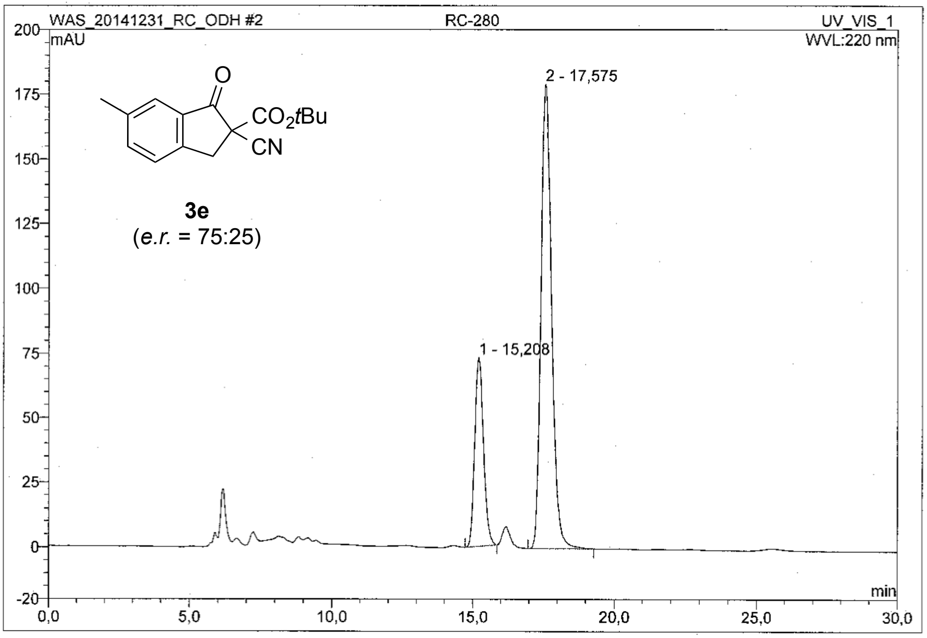

No.	Ret.Time min	Peak Name	Height mAU	Area mAU*min	Rel.Area %	Amount	Type
1	15,21	n.a.	73,085	26,232	25,21	n.a.	BMB
2	17,58	n.a.	179,314	77,827	74,79	n.a.	BMB
Total:			252,399	104,059	100,00	0,000	

Chromeleon (c) Dionex 1996-2006
Version 6.80 SR12 Build 3578 (207169)

4 RC-281

Sample Name:	**RC-281**	*Injection Volume:*	**10,0**
Vial Number:	**RA4**	*Channel:*	**UV_VIS_2**
Sample Type:	**unknown**	*Wavelength:*	**250**
Control Program:	**OD_H_30Min_9_1_flow05**	*Bandwidth:*	**n.a.**
Quantif. Method:	**OD_H**	*Temperature/Column:*	**10**
Recording Time:	**31.12.2014 17:07**	*Flow ml/min:*	**0,500**
Run Time (min):	**30,00**	*Sample Amount:*	**1,0000**

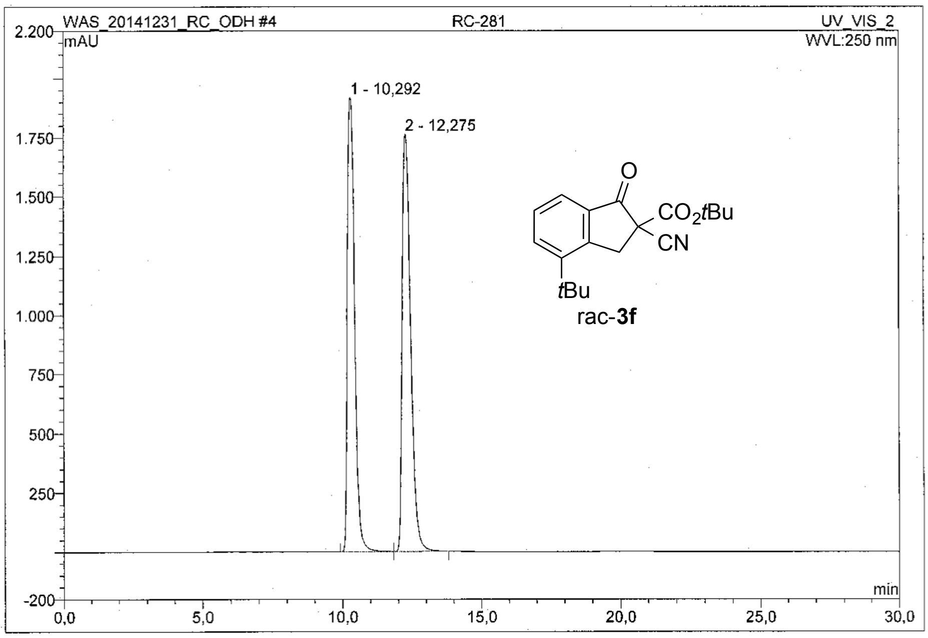

No.	Ret.Time min	Peak Name	Height mAU	Area mAU*min	Rel.Area %	Amount	Type
1	10,29	n.a.	1918,356	579,269	47,72	n.a.	BM
2	12,28	n.a.	1763,170	634,578	52,28	n.a.	MB
**Total:**			3681,526	1213,847	100,00	0,000	

5 RC-282

Sample Name:	**RC-282**	*Injection Volume:*	**10,0**
Vial Number:	**RA5**	*Channel:*	**UV_VIS_2**
Sample Type:	**unknown**	*Wavelength:*	**250**
Control Program:	**OD_H_30Min_9_1_flow05**	*Bandwidth:*	**n.a.**
Quantif. Method:	**OD_H**	*Temperature/Column:*	**10**
Recording Time:	**31.12.2014 17:38**	*Flow ml/min:*	**0,500**
Run Time (min):	**28,58**	*Sample Amount:*	**1,0000**

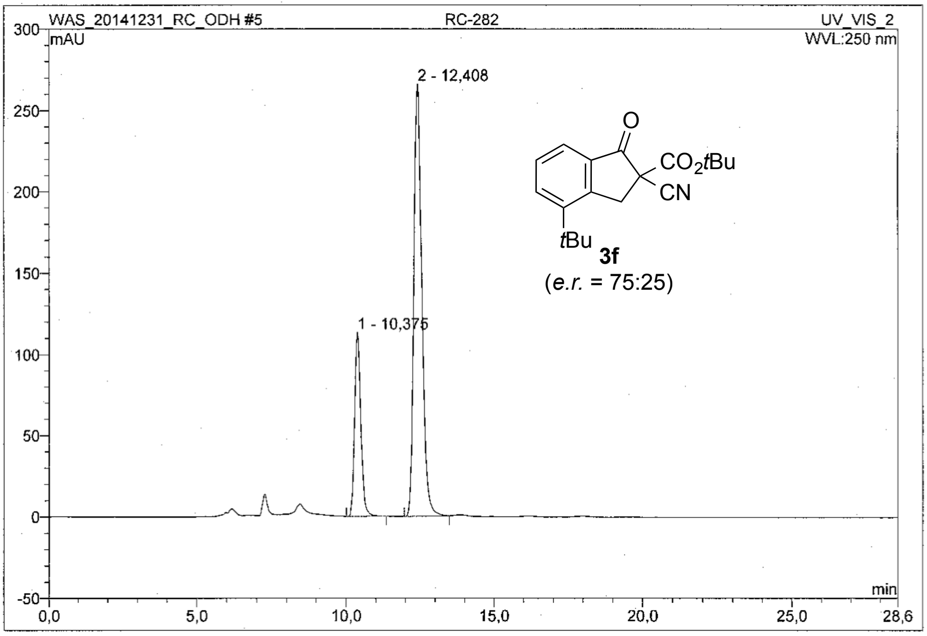

No.	Ret.Time min	Peak Name	Height mAU	Area mAU*min	Rel.Area %	Amount	Type
1	10,38	n.a.	113,483	27,730	25,01	n.a.	BMB
2	12,41	n.a.	265,909	83,143	74,99	n.a.	BMB
**Total:**			379,393	110,873	100,00	0,000	

1 RC-277

Sample Name:	**RC-277**	*Injection Volume:*	**10,0**
Vial Number:	**RA1**	*Channel:*	**UV_VIS_1**
Sample Type:	**unknown**	*Wavelength:*	**n.a.**
Control Program:	**AD_H_60Min_9_1_flow080**	*Bandwidth:*	**n.a.**
Quantif. Method:	**AD_H**	*Dilution Factor:*	**1,0000**
Recording Time:	**3.1.2015 17:55**	*Sample Weight:*	**1,0000**
Run Time (min):	**31,90**	*Sample Amount:*	**1,0000**

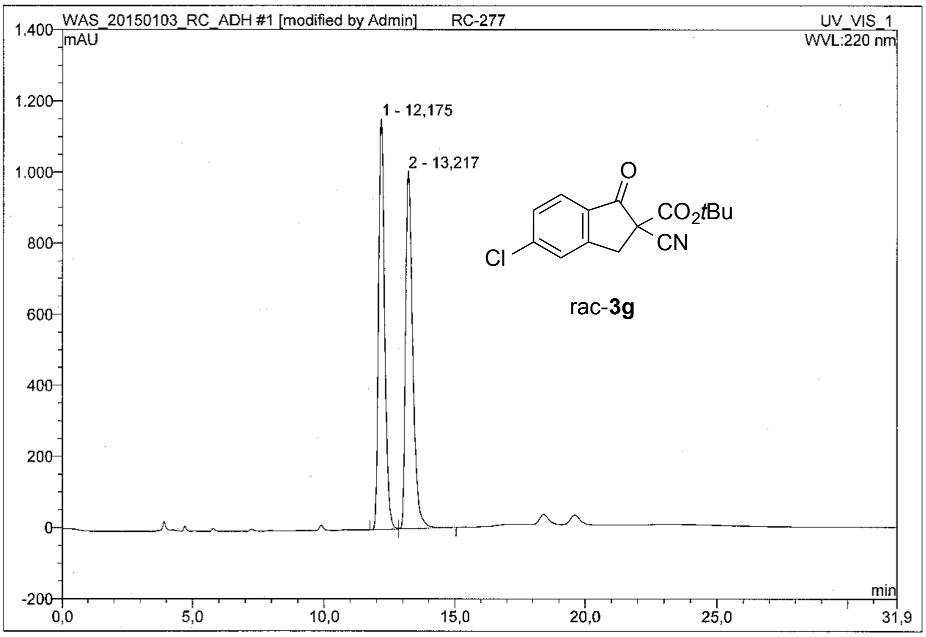

No.	Ret.Time min	Peak Name	Height mAU	Area mAU*min	Rel.Area %	Amount	Type
1	12,18	n.a.	1154,629	313,497	49,24	n.a.	BM
2	13,22	n.a.	1005,741	323,112	50,76	n.a.	MB
**Total:**			2160,370	636,609	100,00	0,000	

2 RC-278

Sample Name:	**RC-278**	*Injection Volume:*	**10,0**
Vial Number:	**RA2**	*Channel:*	**UV_VIS_1**
Sample Type:	**unknown**	*Wavelength:*	**n.a.**
Control Program:	**AD_H_60Min_9_1_flow080**	*Bandwidth:*	**n.a.**
Quantif. Method:	**AD_H**	*Dilution Factor:*	**1,0000**
Recording Time:	**3.1.2015 18:29**	*Sample Weight:*	**1,0000**
Run Time (min):	**36,72**	*Sample Amount:*	**1,0000**

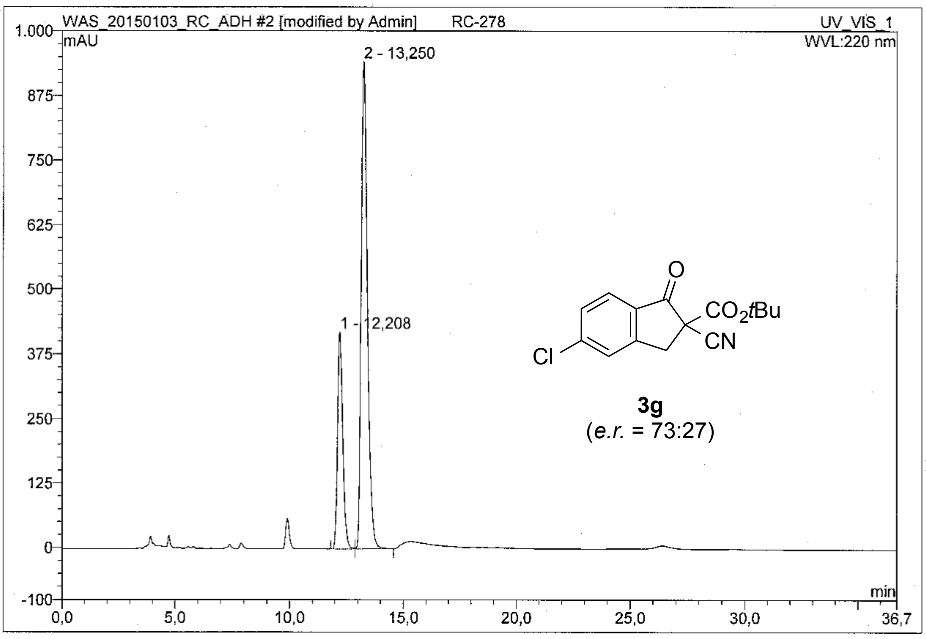

No.	Ret.Time min	Peak Name	Height mAU	Area mAU*min	Rel.Area %	Amount	Type
1	12,21	n.a.	419,932	110,943	27,11	n.a.	BM
2	13,25	n.a.	943,421	298,313	72,89	n.a.	MB
**Total:**			1363,353	409,257	100,00	0,000	

1 RC-285

Sample Name:	**RC-285**	*Injection Volume:*	**10,0**
Vial Number:	**RA1**	*Channel:*	**UV_VIS_1**
Sample Type:	**unknown**	*Wavelength:*	**n.a.**
Control Program:	**AD_H_60Min_9_1_flow080**	*Bandwidth:*	**n.a.**
Quantif. Method:	**AD_H**	*Dilution Factor:*	**1,0000**
Recording Time:	**5.1.2015 15:21**	*Sample Weight:*	**1,0000**
Run Time (min):	**18,28**	*Sample Amount:*	**1,0000**

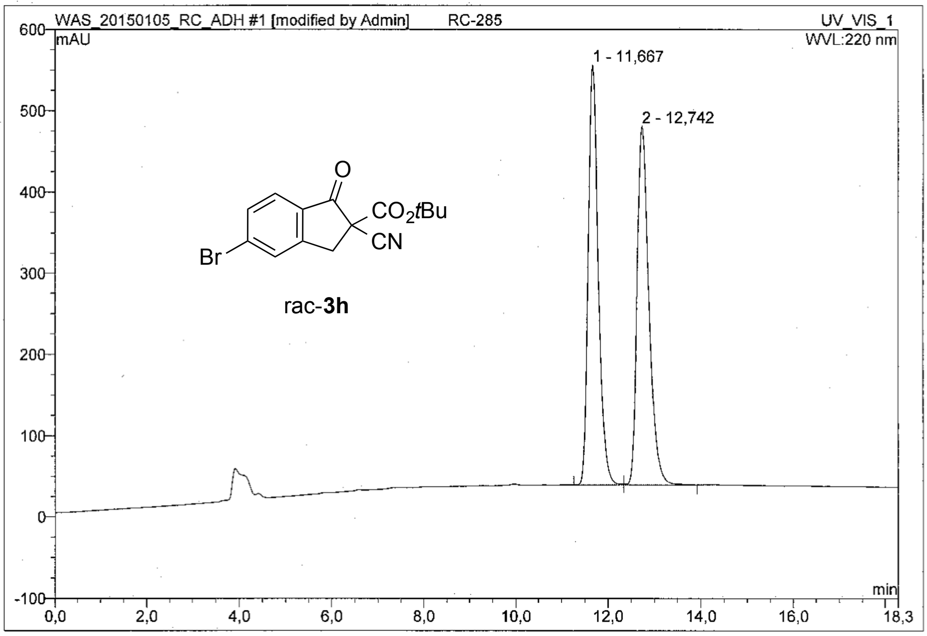

No.	Ret.Time min	Peak Name	Height mAU	Area mAU*min	Rel.Area %	Amount	Type
1	11,67	n.a.	516,767	130,946	49,96	n.a.	BM
2	12,74	n.a.	441,159	131,171	50,04	n.a.	MB
Total:			957,926	262,117	100,00	0,000	

2 RC-286

Sample Name:	**RC-286**	*Injection Volume:*	**10,0**
Vial Number:	**RA2**	*Channel:*	**UV_VIS_1**
Sample Type:	**unknown**	*Wavelength:*	**n.a.**
Control Program:	**AD_H_60Min_9_1_flow080**	*Bandwidth:*	**n.a.**
Quantif. Method:	**AD_H**	*Dilution Factor:*	**1,0000**
Recording Time:	**5.1.2015 15:41**	*Sample Weight:*	**1,0000**
Run Time (min):	**21,18**	*Sample Amount:*	**1,0000**

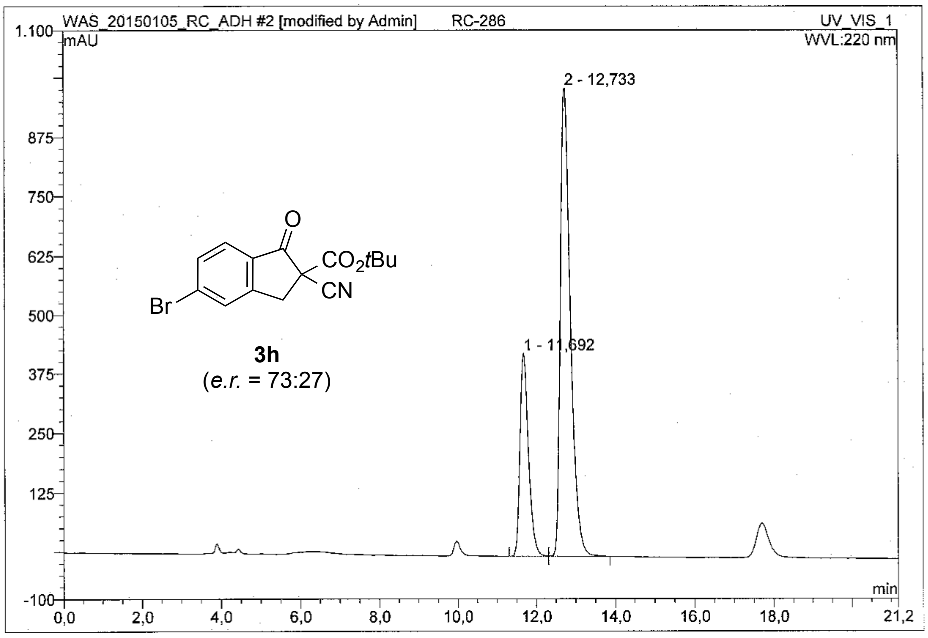

No.	Ret.Time min	Peak Name	Height mAU	Area mAU*min	Rel.Area %	Amount	Type
1	11,69	n.a.	427,560	108,338	26,98	n.a.	BM
2	12,73	n.a.	988,847	293,207	73,02	n.a.	MB
**Total:**			1416,407	401,545	100,00	0,000	

Chromeleon (c) Dionex 1996-2006
Version 6.80 SR12 Build 3578 (207169)
